# Needs-based off-job crafting across different life domains and contexts: Testing a novel conceptual and measurement approach

**DOI:** 10.3389/fpsyg.2022.959296

**Published:** 2022-09-22

**Authors:** Miika Kujanpää, Christine Syrek, Louis Tay, Ulla Kinnunen, Anne Mäkikangas, Akihito Shimazu, Christopher W. Wiese, Rebecca Brauchli, Georg F. Bauer, Philipp Kerksieck, Hiroyuki Toyama, Jessica de Bloom

**Affiliations:** ^1^Faculty of Social Sciences (Psychology), Tampere University, Tampere, Finland; ^2^School of Business, University of South-Eastern Norway, Hønefoss, Norway; ^3^Department of Management Sciences, University of Applied Sciences Bonn-Rhein-Sieg, Rheinbach, Germany; ^4^Department of Psychological Sciences, Purdue University, West Lafayette, IN, United States; ^5^Faculty of Social Sciences (Work Research Centre), Tampere University, Tampere, Finland; ^6^Faculty of Policy Management, Keio University, Kanagawa, Japan; ^7^School of Psychology, Georgia Institute of Technology, Atlanta, GA, United States; ^8^Digitization Initiative of the Zurich Universities, University of Zurich, Zurich, Switzerland; ^9^Public and Organizational Health, Center of Salutogenesis, Epidemiology, Biostatistics, and Prevention Institute, University of Zurich, Zurich, Switzerland; ^10^Department of Education, University of Helsinki, Helsinki, Finland; ^11^Department of HRM & OB, University of Groningen, Groningen, Netherlands

**Keywords:** off-job crafting, leisure crafting, optimal functioning, integrative needs model of crafting, needs satisfaction, DRAMMA model, validation, needs-based

## Abstract

Shaping off-job life is becoming increasingly important for workers to increase and maintain their optimal functioning (i.e., feeling and performing well). Proactively shaping the job domain (referred to as job crafting) has been extensively studied, but crafting in the off-job domain has received markedly less research attention. Based on the *Integrative Needs Model of Crafting*, needs-based off-job crafting is defined as workers’ proactive and self-initiated changes in their off-job lives, which target psychological needs satisfaction. Off-job crafting is posited as a possible means for workers to fulfill their needs and enhance well-being and performance over time. We developed a new scale to measure off-job crafting and examined its relationships to optimal functioning in different work contexts in different regions around the world (the United States, Germany, Austria, Switzerland, Finland, Japan, and the United Kingdom). Furthermore, we examined the criterion, convergent, incremental, discriminant, and structural validity evidence of the Needs-based Off-job Crafting Scale using multiple methods (longitudinal and cross-sectional survey studies, an “example generation”-task). The results showed that off-job crafting was related to optimal functioning over time, especially in the off-job domain but also in the job domain. Moreover, the novel off-job crafting scale had good convergent and discriminant validity, internal consistency, and test–retest reliability. To conclude, our series of studies in various countries show that off-job crafting can enhance optimal functioning in different life domains and support people in performing their duties sustainably. Therefore, shaping off-job life may be beneficial in an intensified and continually changing and challenging working life.

## Introduction

Modern working life is increasingly characterized by flexibility regarding when and how work tasks are performed. Flexible work can promote high permeability between the job and the off-job domains, wherein optimal functioning, defined as feeling and performing well (see also Ryan and Deci, 2001) in one domain, may influence functioning in the other (Field and Chan, 2018; Wepfer et al., 2018). Building on the *Integrative Needs Model of Crafting* (de Bloom et al., 2020), which identifies needs as core elements in the crafting process, we give substance to further developing and assessing the proposed concept of needs-based off-job crafting. Off-job crafting (OJC) is defined as workers’ proactive and self-initiated changes in their off-job lives which target psychological needs satisfaction.

In keeping with the Integrative Needs Model of Crafting (de Bloom et al., 2020), we consider psychological needs to be central to the crafting process. As psychological needs are not restricted to specific life domains, unsatisfied needs in one domain can inspire crafting in other domains, reflecting the mechanism of compensation (Petrou and Bakker, 2016). Moreover, spillover mechanisms may be at play when people apply the same crafting strategies across several life domains to fulfill their needs (Demerouti et al., 2020). As “psychological needs are holistically fulfilled across different [life] domains” (de Bloom et al., 2020, p: 8), job crafting can affect work and non-work outcomes; and OJC can similarly benefit work outcomes as well as non-work outcomes. Accordingly, a better understanding of crafting occurring outside the work domain can enrich the existing large body of research on job crafting and help explain well-being changes occurring in private life and at work.

Our series of five studies has two major aims: First, we further develop and refine the concept of needs-based OJC by building on, extending, and connecting earlier conceptualizations of leisure crafting, home crafting, and psychological needs satisfaction, aligning with the identity-based integrative needs model of crafting (de Bloom et al., 2020). Second, we develop and validate a scale to measure needs-based OJC based on this conceptualization: the *Needs-based Off-job Crafting Scale* (abbreviated as NOCS).

### Prior research on crafting outside the job

While research on job crafting is plentiful (for reviews and meta-analyses: Rudolph et al., 2017; Lichtenthaler and Fischbach, 2019; Zhang and Parker, 2019; Lazazzara et al., 2020), research on crafting outside the work context is scarce. The two primary constructs relevant here are leisure crafting and home crafting.

Firstly, *leisure crafting* has been defined as “exercising initiative, agency and proactivity to create opportunities for experiencing states of enjoyment and meaning” in leisure time (Berg et al., 2010, p: 982). Berg et al. (2010) proposed two specific leisure crafting techniques: hobby participating (pursuing unanswered callings through hobby-related leisure activities) and vicarious experiencing (seeking to fulfill one’s calling through other persons participating in activities related to the unanswered calling). Building on Berg et al.’s (2010) pioneering theoretical work and relying on Wrzesniewski and Dutton (2001) work on job crafting, Petrou and Bakker (2016) developed a scale to measure leisure crafting as proactive efforts to reshape cognitive, task or relational boundaries of leisure and thus focus on a mix of goals and psychological needs that people may address in their crafting efforts. In two empirical studies, they found that workers are more likely to use leisure crafting when they experience high job strain combined with high home autonomy. Weekly leisure crafting was positively related to the satisfaction of the needs for relatedness and autonomy (Petrou and Bakker, 2016) and meaning-making, but only when opportunities for job crafting were scarce (Petrou et al., 2017).

Secondly, *home crafting* (Demerouti et al., 2020) hinges on the conceptual framework of job crafting as proposed by Tims and Bakker (2010). Accordingly, home crafting refers to seeking challenges and reducing demands at home. Aligning with job crafting measures within this research stream, the home crafting questionnaire only assesses concrete behaviors and does not include people’s motivation for these behaviors. Demerouti et al. (2020) reported positive associations between seeking resources at work and seeking resources at home, as well as between seeking challenges at work and at home, suggesting spillover between crafting efforts in the two life domains. Reducing demands at work was negatively related to reducing demands at home, suggesting a compensatory relationship. Relationships with optimal functioning at work or at home were not investigated in this study, leaving the criterion validity questions of the home crafting scale so far unaddressed.

The current contribution of the OJC concept is that it provides a coherent, broad, and yet parsimonious framework for crafting aligning with the identity-based integrative needs model of crafting (de Bloom et al., 2020). Our theoretical model for OJC is firmly rooted in psychological needs transcending and uniting life domains, and integrating people’s crafting motives and efforts. Our OJC concept and the novel integrates research on prior crafting concepts (i.e., job, leisure, and home crafting) and psychological needs and offers opportunities to examine and understand crafting processes occurring within and across various life domains through the same theoretical lens: psychological needs as drivers and needs satisfaction as an outcome of individual crafting efforts. Please see Appendix 1 for an overview of existing crafting concepts, their key characteristics, and distinguishing features. The novel NOCS scale is the first instrument to measure crafting in off-job life including multiple off-job life domains, instead of solely focusing on the leisure or home domain. The NOCS, showing criterion, convergent, incremental, discriminant, and structural validity across various countries and languages, enables researchers to measure OJC as a counterpart and complement of job crafting, thereby arriving at a better understanding of their interrelations and a more complete understanding on how crafting processes outside the job contribute to well-being within and across different life domains.

In the following sections, we introduce the concept of needs-based OJC and a new scale to measure needs-based OJC with the goal of arriving at a coherent and integrative understanding of the crafting efforts people employ across life domains. Then, we present our hypotheses which are tested in a series of five studies among workers from the United States, three German-speaking countries, Finland, Japan, and the United Kingdom.

### Needs-based crafting

The Integrative Needs Model of Crafting (de Bloom et al., 2020) portrays crafting as deliberate strategies aimed at needs satisfaction. These strategies, referred to as “crafting efforts,” may occur at work (i.e., job crafting) or outside work (i.e., OJC) but in both instances address overarching needs that span across life domains. The model posits several key characteristics of crafting: First, crafting is proactive and self-initiated, as opposed to reactive behaviors such as coping, which are a response to stressful situations. Thus, crafting involves conscious thought processes such as planning, goal setting, and problem solving (Baumeister et al., 2011). Second, crafting is intentional and deliberate, and thus distinct from routinely engaging in activities. Third, crafting is self-targeted (e.g., Tims et al., 2012), aimed at satisfying a person’s individual psychological needs. Fourth, crafting is substantial, meaning that crafting concerns mid- or long-term changes in behaviors and cognitions rather than singular or incidental changes. Moreover, the model posits that the main goal of crafting efforts is increasing psychological needs satisfaction, which may ultimately lead to optimal functioning in both off-job and job domains. Taken together, the proactive, self-initiated, intentional, deliberate, and self-targeted nature of needs-based OJC efforts distinguishes the concept from needs satisfaction and recovery experiences, which constitute mental states that are not necessarily acquired through proactive, self-initiated and intentional efforts. Furthermore, based on the two-process model of needs (Sheldon, 2011), the Integrative Needs Model of Crafting distinguishes between needs discrepancy and needs satisfaction (de Bloom et al., 2020). Whereas perceived needs discrepancy constitutes the driver in crafting efforts, needs satisfaction is the experiential reward experienced when crafting efforts are successful.

#### Needs-based off-job crafting

In this study, we focus on needs-based OJC, defined as workers’ proactive and self-initiated changes in their off-job lives targeted at psychological needs satisfaction. In keeping with the Integrative Needs Model of Crafting (de Bloom et al., 2020), we view DRAMMA needs (i.e., detachment, relaxation, autonomy, mastery, meaning, and affiliation) as the main foci of OJC through which workers may (seek to) pursue optimal functioning and well-being (Newman et al., 2014; Kuykendall et al., 2017).

The Integrative Needs Model of Crafting posits that successful OJC efforts targeting needs satisfaction create optimal functioning especially in the same life domain (e.g., life satisfaction and family role performance; de Bloom et al., 2020). Importantly, however, since needs satisfaction constitutes the basis of human behavior and “cannot easily be isolated into a single domain” (de Bloom et al., 2020, p. 9), the effects of OJC efforts can spill over to the work domain. In accordance with the integrated model of human energy by Quinn et al. (2012), we argue that OJC can improve optimal functioning in the job domain as well. Accordingly, needs-based OJC can potentially restore personal resources depleted through work (i.e., a compensation mechanism) and build new resources that can translate to the job domain (Edwards and Rothbard, 2000; Hobfoll, 2001; Quinn et al., 2012; de Bloom et al., 2020). Assuming a compensation mechanism, OJC can restore personal resources such as mental energy depleted by tiring or boring work-related activities (Sonnentag et al., 2017). By proactively engaging in enjoyable, relaxing leisure activities, personal resources can be restored, which can enhance functioning both in the off-job and the work domain (Sonnentag, 2003; Ten Brummelhuis and Bakker, 2012).

OJC efforts may also help to build new resources. For instance, by engaging in challenging hobbies, people may gain new knowledge or skills they may also use at work [see Stebbins (2001) perspective on serious leisure]. In a similar vein, Kelly et al. (2020) demonstrated that engagement in leisure activities could help to build self-efficacy and support sustainable careers. Summing up, OJC can presumably create optimal functioning in both the off-job and the job domain (such as life satisfaction; de Bloom et al., 2020, and work ability; McGonagle et al., 2015). Therefore, the concept of OJC is relevant for both leisure and occupational well-being – and even other non-work life domains.

Needs-based OJC extends past work on crafting in three crucial ways. First, our theorization and operationalization of OJC seek explicitly to include multiple aspects of the non-work life domain so far under-researched in the crafting literature (i.e., leisure, voluntary work, household chores, and childcare, and work breaks), which can potentially be more important than work, because workers typically spend more time outside work than at work. Second, while we posit that OJC efforts are generally targeted at increasing needs satisfaction, we acknowledge that there may be individual differences as to which activities are directed at which specific needs (de Bloom et al., 2020). For example, a person might watch a documentary to relax and distance themselves from pressures at home (i.e., OJC for relaxation), while another person might watch the same documentary to gain new knowledge (i.e., OJC for mastery), and a third person may watch it without any purpose in mind (in which case this behavior would not be proactive and thus not considered crafting). Therefore, we distinguish between different foci of OJC (i.e., OJC for relaxation and OJC for mastery) and emphasize the goal-oriented nature of crafting. In line with research that shows that conscious thought processes are profound and common influencers of behavioral processes and outcomes (Baumeister et al., 2011), we distinguish OJC from engagement in recreational activities, often occurring in a non-deliberative manner (Iso-Ahola, 2015). Third, an explicit theoretical link between crafting goals and psychological needs theories has so far been lacking. Earlier research has often mixed goals and needs in conceptualizations of crafting or measured crafting only in behavioral terms without considering *why* people engage in certain behaviors. By defining crafting goals in terms of needs satisfaction and psychological needs as drivers for actual crafting efforts, our theoretical framework aligns closely with the identity-based integrative needs model of crafting and explains the processes through which crafting enhances optimal functioning off and on the job (de Bloom et al., 2020).

### The present research

In this series of studies, we first developed the Needs-based Off-job Crafting Scale (NOCS) with the help of expert panels, qualitative interviews and two cross-sectional studies examining the factor structure of the scale in exploratory analyses, as well as evidence for criterion validity (Studies 1a-b). Validity for needs-based OJC was then established in five sub-studies in samples of workers from different countries: three studies with repeated measurements in longitudinal data collections each covering a six-month period (in three German-speaking countries in Study 2, Finland in Study 3, and Japan in Study 4) and one cross-sectional study (in the United Kingdom, Study 5). Establishing the validity of needs-based OJC and the NOCS in different countries and working contexts provides more comprehensive validation evidence than single-country designs, which are typically used in validation studies. Moreover, crafting efforts occur dynamically over time (e.g., Petrou et al., 2017; Zhang and Parker, 2019), suggesting that crafting may not be fully captured using only cross-sectional research designs. Thus, conducting studies with repeated measurements in different countries and languages provides a more complete and robust validation of needs-based OJC. Next, we present hypotheses concerning five aspects of validity of OJC examined in this study, that is, criterion, convergent, incremental, discriminant, and structural validity evidence.

#### Criterion validity evidence

As first evidence of criterion validity, we examined if workers can give concrete examples for their crafting efforts for all six OJC dimensions, and if the number of examples matches people’s scores on the newly developed scale.

*Hypothesis 1*: The number of examples for OJC efforts correlates positively with the matching scale dimension of the NOCS (Study 1b).

A second important test regarding the criterion validity is whether OJC targeted at proactively increasing needs satisfaction is positively related to needs satisfaction over time. In keeping with the Integrative Needs Model of Crafting (de Bloom et al., 2020), we expect that OJC aiming to satisfy a certain need would have a positive lagged effect on the satisfaction of that need.

*Hypothesis 2*: OJC targeted at satisfying a DRAMMA need at T1 is positively related to the satisfaction of that need at T2 and T3 (Study 3).

In their review, Newman et al. (2014) conceptualized the DRAMMA needs as core mechanisms linking leisure to better subjective well-being. Several studies have reported positive relationships between leisure time DRAMMA needs satisfaction and life satisfaction (e.g., Park and Fritz, 2015; Walker and Kono, 2018; Kujanpää et al., 2020). Consequently, OJC should be related to improved well-being in the off-job domain through increased needs satisfaction (Newman et al., 2014; Sirgy et al., 2017; de Bloom et al., 2020). Thus, we expect OJC to be positively associated with life satisfaction over time.

Family role performance refers to “the fulfillment of obligations and expectations stemming from the roles associated with participation in the family domain” (Chen et al., 2014, p: 193). It is a theoretical equivalent to work role performance in the home domain, comprising two factors: task and relational (contextual) performance in family roles (Chen et al., 2014). Since OJC is initiated with the purpose of aligning one’s off-job life with personal needs, it may help workers to optimize and better perform family-related tasks and relationships. Thus, OJC should be positively related to family role performance.

Since needs satisfaction has positive effects in different life domains (Milyavskaya and Koestner, 2011; de Bloom et al., 2020), successful OJC efforts may be related to well-being at work through congruence and spillover effects (e.g., Edwards and Rothbard, 2000; Sonnentag and Kühnel, 2016; Walker and Kono, 2018). Increased personal resources such as vigor and positive affect gained through needs satisfaction in off-job life can positively predict not only off-job life-related but also work-related well-being (e.g., Hecht and Boies, 2009; Fritz and Demsky, 2019; Sirgy et al., 2020). Thus, we expect OJC also to be positively related to job satisfaction over time.

Perceived work ability, on the other hand, refers to a worker’s perceived ability to meet the demands of the job (Cadiz et al., 2019). OJC can provide workers with personal resources such as autonomy and mastery that are critical for work ability (McGonagle et al., 2015; Cadiz et al., 2019). Thus, in accordance with the Integrative Needs Model of Crafting (de Bloom et al., 2020), OJC may enrich personal resources that can be translated to the job domain, helping workers to face challenges at work and benefiting their work ability.

Moreover, we expect OJC to be positively associated with work engagement over time. Work engagement is driven by positive emotions generated by needs satisfaction (Green et al., 2017). Proactively increasing needs satisfaction through OJC could help to energize workers not only in their off-job life but also at work. In line with the model of human energy (Quinn et al., 2012) and research showing that detachment from work, relaxation, and mastery during leisure time can energize work engagement (de Bloom et al., 2015; Sonnentag and Kühnel, 2016), needs-based OJC has thus potential to enhance work engagement.

*Hypothesis 3a-e*: OJC at T1 is positively related to optimal functioning at T2 and T3; more specifically to (a) life satisfaction (Studies 2-4), (b) family role performance (Studies 3-4), (c) job satisfaction (Studies 2-4), (d) perceived work ability (Studies 3-4) and (e) work engagement (Studies 2-4).

#### Convergent and incremental validity evidence

As job crafting is a well-researched phenomenon and other types of crafting outside work have been proposed and studied, we examined whether OJC is (1) positively related to and (2) predicts optimal functioning in the off-job (life satisfaction, family role performance) and the job domain (job satisfaction, perceived work ability, work engagement) *beyond* job crafting, proactive personality and the recently developed constructs of leisure crafting and home crafting.

Earlier crafting research lacked a coherent framework to explain and predict crafting efforts both at work and outside work (de Bloom et al., 2020). Most job crafting research is grounded on the job demands-resources model (Demerouti et al., 2001), which is difficult to translate to the non-work context, which potentially offers a much wider variety of demands and resources than the work context. Moreover, people’s crafting motivation remains unclear when using purely behavioral definitions of job crafting efforts (e.g., asking for help), making it questionable if and for what aspect people are crafting (i.e., is “asking for help” increasing resources or lowering demands?). Thus, we expect OJC to have incremental value in predicting optimal functioning over and above job crafting.

Regarding personality and crafting, it has been shown that people with a proactive personality tend to craft more (e.g., Bakker et al., 2012). OJC requires a proactive, bottom-up stance for individuals to self-manage their off-job lives with the aim of satisfying personal needs. Therefore, we expect that individuals with more proactive personalities, i.e., those who are relatively unaffected by situational forces and inclined to be actors of environmental change (Bateman and Crant, 1993), would tend to craft their off-job lives more than less proactive individuals. Still, we expected that it does make a difference for optimal functioning whether workers engage in OJC efforts, regardless of their general tendencies to act proactively. Thus, we expect that OJC predicts optimal functioning beyond proactive personality.

Moreover, we posit that OJC is a broader concept than leisure crafting and home crafting and includes different types of crafting efforts entirely absent from leisure crafting and home crafting (such as crafting for detachment, for relaxation, and for meaning). Compared to leisure and home crafting, OJC involves more off-job life domains (such as voluntary work, house- and childcare). In addition, similar to job crafting, home crafting only involves concrete behaviors with no regard to the motivation of these behaviors. Thus, we expect OJC to predict additional variance in optimal functioning beyond the effects of leisure crafting and home crafting.

*Hypothesis 4a-d*: OJC is positively related to (a) job crafting (Study 5), (b) proactive personality (Study 3), (c) leisure crafting (Study 3), and (d) home crafting (Study 5).

*Hypothesis 5a-d*: OJC predicts variance in optimal functioning beyond (a) job crafting (Study 5), (b) proactive personality (Study 3), (c) leisure crafting (Study 3) at T1, and (d) home crafting (Study 5).

#### Discriminant validity evidence

OJC specifically concerns proactive and self-initiated changes in off-job life which target needs satisfaction. Thus, crafting is distinct from simply taking part in recreational activities. While crafting is implemented *via* different off-job behaviors and cognitions, often manifesting through various leisure activities such as starting a new hobby to improve mastery or taking a hot bath to relax, those activities are individually targeted at satisfying different needs according to personal preferences and goals. Moreover, some activities may be routine or externally regulated and thus cannot be categorized as crafting (de Bloom et al., 2020). Thus, we expect that when examined conjointly with OJC, recreational activities would be distinct from OJC, demonstrating discriminant validity evidence between the two constructs (Study 3).

*Hypothesis 6*: Engaging in recreational activities (e.g., resting, volunteering, exercising) is distinct from OJC (Study 3).

#### Structural validity evidence

We examined the factorial structure, internal consistency, and test–retest reliability as indicators of structural validity of the NOCS. In Study 1a, we developed the NOCS and examined its factorial structure and internal consistency. Furthermore, we reduced the total number of scale items from 36 to 18 based on the results, qualitative interviews, and theoretical clarity. We expected to find a six-factor structure for the NOCS based on the DRAMMA model (Newman et al., 2014), which guided our scale development process (presented in detail in Study 1a).

Next, we examined the structural validity evidence of the six-factor solution, i.e., the final model based on the results from Study 1a, in three sub-studies with repeated measurements in the same persons (Studies 2–4). In Study 2, we first tested the six-factor model against other factorial solutions. Alternative models were a one-factor model with all the OJC dimensions loading on a single factor (Petrou and Bakker, 2016), a two-factor model with crafting for detachment and crafting for relaxation loading on the first factor and the rest of the six dimensions on the second factor, and a five-factor model with crafting for detachment and crafting for relaxation loading on the first factor and the other dimensions loading on their unique factors. The choice of the two- and five-factor structures tested was guided by the literature stating that detachment and relaxation are “passive recovery” or “pre-recovery” mechanisms, whereas the other factors may constitute “active recovery” mechanisms (Ten Brummelhuis and Trougakos, 2014). Furthermore, detachment and relaxation correlated highly with each other (Bennett et al., 2018) and were conceptualized as constituting a single factor by Newman et al. (2014), which is why we considered it important also to test the five-factor solution. Based on the results of Study 1a, supporting a six-factor model, and because we expected that crafting efforts focused on the six DRAMMA needs would be distinguishable from one another, we expected that the six-factor model would have a better fit than alternative models.

Moreover, we tested whether the six-factor solution would show invariance over time in different countries (Studies 2–4). As a measure of stability and a further indicator of structural validity evidence, we also examined test–retest reliability of the NOCS in Study 2.

## Study 1a: Scale development and explorative analysis

The goal of Study 1a was to develop a new scale (the NOCS) to measure needs-based OJC, and to examine the factorial structure and internal consistency of the NOCS.

### Scale development

We used a deductive, theory-driven approach in scale development. In deductive scale development, the definition of the construct under measurement guides item development (Hinkin, 1998). We analyzed existing crafting instruments and instruments measuring elements of the DRAMMA model (Newman et al., 2014). Multiple group discussions among the authors were organized to categorize, define, and adapt these items and to create new crafting items aiming to capture the essential qualities within the six OJC dimensions based on the DRAMMA needs (Newman et al., 2014), namely *OJC for*: (1) *detachment*, (2) *relaxation*, (3) *autonomy*, (4) *mastery*, (5) *meaning,* and (6) *affiliation*.

We generated the initial item pool on the basis of two criteria: first, each item had to be about behaviors and cognitions targeted at increasing satisfaction of one of the DRAMMA needs (e.g., increasing detaching from work). Second, items had to describe concrete efforts with the goal of satisfying needs (e.g., making sure to relax during off-job time). After careful examination of all the items developed, an expert panel consisting of three authors selected 36 items for qualitative testing, with six items for each of the six OJC dimensions.

To obtain more information about the comprehensibility, readability, content, and face validity evidence of the items, we conducted 21 qualitative interviews with workers in two countries (15 in Finland and 6 in Japan) to gain insights from both Western and Eastern cultural contexts. To increase participant diversity, interviewees were recruited *via* the authors’ professional contacts and with students’ snowball sampling, thereby including a variety of occupations. Participants provided feedback on problematic or unclear items, and on how they understood the concept of needs-based OJC based on the scale. We also asked participants to provide concrete examples for crafting their off-job lives for each dimension to check whether the scale and the concept of crafting were understandable. Participants were able to provide examples for OJC, with a wide variety of examples given for each OJC dimension. Based on the interviews and further discussions within the team of authors, we clarified some of the items and added examples of crafting to the introduction (see Table 1). The response options for the items ranged from 1 (“never”) to 5 (“very often”).

**Table 1:** EFA Factor Structure in Study 1 with the 36-item Version of the NOCS.

	Item	M	SD	1	2	3	4	5	6
**OJC for detachment**	**I’ve made sure to detach from work-related thoughts during off-job time.**	3.93	1.01	0.79					
	**I’ve arranged my off-job time so that I distance myself from work-related tasks.**	3.84	1.05	0.79					
	I’ve planned my off-job activities so that I mentally disengage from my job demands.	3.91	0.96	0.59					
	I’ve made sure to focus my attention on non-work-related matters during off-job time.	4.02	0.90	0.85					
	**I’ve organized my off-job activities so that I switch off from work duties.**	3.81	1.06	0.83					
	I’ve arranged my off-job activities so that I leave job-related issues behind.	3.95	1.03	0.95					
**OJC for relaxation**	**I’ve made sure to experience relaxation of my body and mind during off-job time.**	3.71	0.96		0.98				
	I’ve arranged my off-job activities so that I physically and mentally unwind during off-job time.	3.84	0.98		0.68				
	**I’ve planned my off-job activities so that I get relief from stress.**	3.84	0.99		0.89				
	I’ve organized my off-job time so that I feel at ease.	3.87	0.92		0.68				
	**I’ve arranged my off-job time so that I get some rest.**	3.97	0.98		0.71				
	I’ve made sure to calm down physically and mentally during off-job time.	3.92	0.94		0.74				
**OJC for autonomy**	I’ve made sure to experience autonomy during off-job time.	3.84	1.00			0.56			
	I’ve arranged my off-job time so that I achieve a sense of freedom in the things I undertake.	3.94	0.90		0.36	0.34			
	I’ve planned my off-job activities so that I experience choice in my schedules.	3.74	1.02			0.57			
	**I’ve planned my off-job activities so that I experience control over my life.**	3.97	0.90			0.60			
	**I’ve organized my off-job activities so that I determine my own course of action.**	4.04	0.87			0.61			
	**I’ve made sure that the things I do during off-job time reflect what I really want.**	4.05	0.89			0.48			
**OJC for mastery**	I’ve made sure to feel competent in the things I do during off-job time.	3.68	1.12				0.65		
	I’ve organized my off-job activities so that I develop my skills and abilities.	3.30	1.11				0.93		
	**I’ve arranged my off-job time so that I experience proficiency in the things I undertake.**	3.39	0.99				0.73		
	**I’ve made sure to familiarize myself with new ideas, expand my knowledge or interests during off-job time.**	3.46	1.04				0.81		
	I’ve planned off-job activities to challenge myself.	3.05	1.06				0.68		
	**I’ve organized my off-job activities so that I put my skills, knowledge or abilities into action.**	3.30	1.03				0.83		
**OJC for meaning**	**I’ve made sure to experience meaning in my life during off-job time.**	3.56	1.02					0.70	
	I’ve arranged my off-job time so that I experience value and worth in my activities.	3.65	0.98					0.59	
	**I’ve organized my off-job activities so that I achieve a sense of purpose in what I am doing.**	3.52	1.03					0.73	
	I’ve made sure to focus on what is personally important to me during off-job time.	3.95	1.00					0.85	
	**I’ve arranged my off-job time so that the things I do align with my personal values.**	3.81	1.05					0.66	
	I’ve planned my off-job activities to be reflective of the person I am.	3.83	1.09					0.72	
**OJC for affiliation**	**I’ve made sure to experience close connections to the people around me during off-job time.**	3.67	1.02						0.95
	**I’ve arranged my off-job time so that I feel a sense of belongingness to my family and/or friends.**	3.73	1.00						0.85
	**I’ve planned my off-job activities so that I feel related to those around me.**	3.73	1.05						0.87
	I’ve made sure to create strong social ties within my community during off-job time.	3.01	1.27				0.41		
	I’ve organized my off-job activities so that I connect with persons that are important to me.	3.77	1.05						0.93
	I’ve planned my off-job time so that it brings me, my family, friends or colleagues closer together.	3.65	1.06						0.79

US participants (*n* = 99). Items in bold face selected for the final version of the NOCS. Loadings > 0.32 shown in the table. Response options were 1 (“never”), 2 (“rarely”), 3 (“sometimes”), 4 (“often”) and 5 (“very often”). OJC = off-job crafting.

*Off-job crafting* is about big or small changes persons can make for their non-work time to meet their own goals. It refers to tailoring one’s recreational activities such as hobbies, sports, or travel to fulfill one’s personal needs. Off-job crafting can also include adjustments of work break activities, domestic or (child-) care tasks. Examples for off-job crafting include taking a hot shower in order to relax, learning a foreign language to develop new skills, volunteering to connect with the local community or listening to music to forget about work. Some people actively adjust their off-job activities or thoughts to meet their personal goals and needs, and others do not. How often have you engaged in *off-job crafting* during the past month to meet your own goals? Over the past month, …

The 36-item version of the NOCS was developed in English, and the scale was translated into other languages and back-translated by linguistic experts. We chose a time frame (1 month) for the introduction of OJC items to increase measurement specificity and to facilitate discerning the potential changes in crafting over time. Next, we examined the factor structure of the 36-item version of the NOCS among US workers.

### Methods

Data were collected *via* Amazon Mechanical Turk (MTurk) in July 2018. Participants were paid $4 for participation, and were required to work at least 25 h to be able to participate. In total, 99 participants completed the online questionnaire. One participant who failed the five attention checks used in the survey was removed from the data, leaving a total of 98 participants in the sample. Of the participants, 41% were female, and they were on average 38 years old (*SD* = 9.68). They worked in various occupations, such as accountant, IT manager, electrician, and art director. Participants worked on average 42.8 h weekly (range 25–65). We conducted exploratory factor analysis (EFA) with principal axis factoring (Promax rotation) and Kaiser’s K1 rule and Velicer’s MAP test (O’Connor, 2000) to extract factors. A criterion value of 0.32 was used to retain items within each factor (Costello and Osborne, 2005).

### Results

The means of OJC items ranged from 3.01 to 4.05, and the standard deviations from 0.87 to 1.27 (Table 1). Thus, on average, participants reported that they crafted their off-job life from “sometimes” to “often.” Bartlett’s test of sphericity (*p* < 0.001) and the Kaiser-Meyer-Olkin (KMO) measure of sampling adequacy (0.88) indicated that conducting a factor analysis was appropriate. All extracted item communalities were above 0.50. Kaiser’s K1 rule and Velicer’s MAP test (O’Connor, 2000) both indicated a six-factor solution, supporting the theorized six-factor structure of the NOCS. The six factors explained a total of 76.3% of variance. All items loaded on their theorized factors, except the item “…I’ve made sure to create strong social ties within my community during off-job time,” which loaded on crafting for mastery and not on crafting for affiliation, and the item “…I’ve arranged my off-job time so that I achieve a sense of freedom in the things I undertake,” which loaded on both crafting for autonomy and on crafting for relaxation. In addition, the item “…I’ve made sure that the things I do during off-job time reflect what I really want” had a low loading (0.48) on its theorized factor, crafting for autonomy. All other items had a loading of >0.50 (Table 1). All six dimensions of the 36-item scale version demonstrated high reliability, with Cronbach’s α ranging from 0.90 to 0.95.

In the next step, we reduced the number of items from 36 to 18 (selecting three items for each of the six factors) to improve the usability of the NOCS and to reduce the participant burden (please see Table 1, bolded items for the final 18-item NOCS used in studies 1b-5). We removed the two items which did not load on their theorized factors. Item selection was based conjointly on the factor loadings, insights from the qualitative interviews, and theoretical clarity. As a first test of the functionality of the shortened scale, we repeated the EFA with the shortened scale consisting of 18 items. Although Eigenvalues of factors 4 to 6 did not reach 1, all items loaded (>0.50) only on their respective factors. To summarize the results of Study 1a, the EFA supported the six-factor structure and reliability of the NOCS.

## Study 1b: Criterion validity evidence

In Study 1b, we examined the criterion validity evidence of OJC (Hypothesis 1) using an idea generation task among 97 US workers recruited *via* Prolific. Workers had to work at least 21 h per week to be eligible for participating. Data were collected in June 2021. Participants received £1.88 (approx. $2.50) for participating. Mean age was 31.6 (SD: 7.9), the sample worked on average 41.4 h per week (*SD* = 6.7; range 24–60), 70% had a bachelor’s or higher degree, a minority (11%) were manual workers, and the majority (89%) were white-collar workers working in a variety of sectors. Half of the sample was female (54%).

We examined criterion validity evidence by first assessing mean levels of crafting on each dimension and correlating this score to the number of examples provided. Specifically, we asked, “Please give examples of how you have engaged in off-job crafting in the ways described as in the questions above. Please give as many examples as you can.” As we found that the time taken to respond to the questionnaire correlated significantly with the total number of examples provided (*r* = 0.24; *p* < 0.05), we used partial correlations to control for this effect.

Means of OJC for mastery and for meaning were slightly lower than OJC for relaxation and affiliation. The number of examples generated varied between 1.8 (OJC for meaning) and 4.6 (OJC for detachment). Participants generated more examples for OJC for detachment and for relaxation and fewer examples for OJC for meaning and for affiliation. Typical examples given for OJC for detachment were: hobbies, physical exercise, reading, listening to music, socializing with friends, household activities, and active strategies to separate work and private life, such as scheduling hobbies right after work hours, ignoring emails, leaving work phone/laptop at the office, engaging in only enjoyable activities. OJC for relaxation included examples such as: self-care activities, massages, spa visits, yoga, meditation, deep breathing exercises, stretching, napping and sleeping in, and exercising. OJC for autonomy was realized with the help of goal setting, active planning/scheduling of activities and prioritizing. Examples for OJC for mastery were: learning activities (e.g., skills, about a topic), volunteering for good causes or paid side projects. OJC for meaning was realized *via* time spent with family and friends, reflection on and engagement in value congruent activities, political engagement, religious, and spiritual activities. Typical examples for OJC for affiliation were: “quality time,” meals and activities together with family and friends (e.g., going to the zoo, couple’s massage, dance classes, date night, going out for a drink, playing games). Connecting with family and friends digitally (*via* email, social media, video calling) was also mentioned frequently.

Partial correlations between OJC and the number of examples were: 0.17 (detachment; *p* > 0.05), 0.25 (relaxation and affiliation; *p* < 0.05), 0.28 (mastery; *p* < 0.01), 0.40 (autonomy; *p* < 0.001) and 0.43 (meaning; *p* < 0.001) with an average correlation coefficient of 0.36 across all OJC dimensions. The higher participants scored on any OJC dimension, the more examples they provided for activities they typically engaged in when crafting for the respective dimension, demonstrating criterion validity evidence and supporting Hypothesis 1.

## Study 2: Structural and criterion validity evidence in data with three repeated measurements

In Study 2, we examined structural and criterion validity evidence of OJC (Hypotheses 3a, 3c, 3e) with data collected in three German-speaking countries (Germany, Austria, and Switzerland).

### Methods

We conducted a study with three measurement points in three German-speaking countries (Germany, Austria, and Switzerland). Data were collected between December 2018 and June 2019. In line with the Integrative Needs Model of Crafting, which posits that crafting involves mid- or long-term changes in behaviors and cognitions as opposed to changes that are only incidental or singular (de Bloom et al., 2020), we used three-month time lags between measurement points. Three-month time lags are commonly used in job crafting studies (e.g., Vogt et al., 2016; Sakuraya et al., 2020). Moreover, a three-month interval between measurements responds to Dormann and Griffin’s (2015) call for shortitudinal study designs (i.e., designs that are shorter than 1 year), which should be used especially when developing new constructs for which an optimal time lag cannot be defined empirically from prior data. In total, a convenience sample of 3,232 workers were invited to participate in the study through an online panel data service (Respondi). Workers who worked 19 h or less per week were excluded from the study. Furthermore, partial responders and workers who answered the questionnaire in less than 12 min were excluded. A total of 2,104 workers’ (65%) responses were retained, of which 1,161 workers (55%) participated in all three waves. Slightly over half (52%) of the participants were male. Mean age was 45 (*SD* = 10.56), and 49% had a college or higher-level qualification. Participants worked in various fields, such as in health and social services, public administration, and manufacturing. Most of the participants worked either between 30 and 39 (38%) hours or between 40 and 49 (46%) hours per week. Nonresponse analysis showed that responders who participated in all of the three waves were slightly more often male [52% vs. 48%, *χ*^2^(1) = 4.05, *p* < 0.05], older [mean age 45 years vs. 42 years, *t*(2102) = 8.02, *p* < 0.01], and had proportionally more workers who worked 30–39 h [41% vs. 35%, *χ*^2^(3) = 9.50, *p* < 0.05] as compared to responders who participated in only one and two waves.

The 18-item version of the NOCS developed in Study 1a was used at all three time points to measure OJC over the past month (Table 1, items in bold face). Cronbach’s α ranged from 0.85 to 0.87 for crafting for detachment, from 0.81 to 0.83 for crafting for relaxation, from 0.71 to 0.75 for crafting for autonomy, from 0.77 to 0.80 for crafting for mastery, from 0.74 to 0.77 for crafting for meaning, and from 0.86 to 0.88 for crafting for affiliation, demonstrating adequate reliability for the scale dimensions.

Life satisfaction and job satisfaction were measured at all-time points with single-item measures adapted from Van den Broeck et al. (2010). Using single-item measures for life and job satisfaction is a valid practice when the interest is in the general satisfaction (Wanous et al., 1997; Lucas and Donnellan, 2012). The items were “How satisfied are you when you look at your private life as a whole?” for life satisfaction, and “How satisfied are you when you look at your professional life as a whole?” for job satisfaction. The scale for these items ranged from 1 (“extremely dissatisfied”) to 7 (“extremely satisfied”). Work engagement was measured at all-time points with the vigor and dedication subdimensions of the Utrecht Work Engagement Scale-9 (six items in total; Schaufeli et al., 2006), which are considered the key dimensions of work engagement (González-Romá et al., 2006). A sample item for work engagement is “At my job, I felt strong and vigorous.” The scale for work engagement ranged from 0 (“never”) to 6 (“always”) and Cronbach’s α ranged from 0.94 to 0.95.

We used longitudinal confirmatory factor analysis to test the structural validity evidence of the NOCS. First, we compared the fit of the six-factor solution of the NOCS from Study 1a with alternative models (i.e., one-factor, two-factor, and five-factor model). Second, to assess whether the respective indicators of the OJC dimensions represent the same underlying construct over time, we followed a step-by-step approach recommended by Little et al. (Little et al., 2007; Little, 2013). In this approach, each tested model is more constrained than its predecessor, representing different degrees of invariance. The goal of this approach is therefore to test whether the indicators of the OJC dimensions measure OJC in the same way across time. Before modeling, we tested the data for multivariate normal distribution with the package MVN in R (Korkmaz et al., 2014). The Mardia test indicated that the data were not multivariate normally distributed. Therefore, we used robust maximum likelihood as an estimator and present robust fit indicators. Longitudinal confirmatory factor analyses were conducted using the package lavaan in R (Rosseel, 2012). We started modeling with an unconstrained model (configural invariance). In the next step, we tested a model in which the loadings of the corresponding indicators are equated across time (loading invariance). In the third step, we tested intercept and residual invariance (equating intercepts and residual variances of the corresponding indicators across time). Model fit was examined by comparing goodness-of-fit indicators (TLI, CFI, RMSEA and SRMR; Hu and Bentler, 1999; Schermelleh-Engel et al., 2003). For model comparison, we examined ∆*χ*^2^ and ∆*χ*^2^*df* to assess whether adding additional constraints was justifiable.

Test–retest reliability was assessed with zero-order Pearson’s correlations within each OJC dimension over time. For criterion validity evidence, we examined partial correlations between each OJC dimension at T1 and each outcome at T2 and T3. Baseline (T1 measurement) of each outcome was controlled for in all partial correlation analyses.

The Harman single-factor test was computed to check for common method bias. The results showed that the obtained single factor accounted for 30.0% of the total variance, indicating that common method bias was not present.

### Results

First, we assessed whether a one-factor model, a two-factor model (DR vs. AMMA) or a five-factor model with crafting for detachment and for relaxation on a single factor led to a better fit than the six-factor model. The results indicated that the fit of a model with one factor (Δ*χ*^2^ = 8914.999, Δ*df* = 112, *p* < 0.001) was worse than the six-factor model (Table 2). A model with two factors (crafting for detachment and for relaxation on one factor, the other dimensions on the other factor) also led to a worse fit compared to the six-factor model (Δ*χ*^2^ = 4404.665, Δ*df* = 109, *p* < 0.001). Moreover, the fit for the five-factor model was worse than the six-factor model (Δ*χ*^2^ = 1008.524, Δ*df* = 88, *p* < 0.001). To summarize, fit of the six-factor model was superior compared to the alternative models.

**Table 2:** Alternative models tested (Study 2) and measurement invariance over time (Studies 2–4).

	*χ* ^2^	*df*	*p*	R-RMSEA (90% CI)	R-CFI	R-TLI	SRMR
**Study 2 (German, Austrian, and Swiss participants, *n* = 2,105)**							
Alternative tested factor structures of the NOCS							
One-factor model	9813.094	472	<0.001	0.138 [0.135; 0.140]	0.677	0.686	0.103
Two-factor model	5302.760	469	<0.001	0.099 [0.097; 0.101]	0.835	0.838	0.071
Five-factor model	1906.619	448	<0.001	0.055 [0.053; 0.058]	0.951	0.950	0.041
Measurement invariance (six-factor model)							
Configural invariance	898.095	360	<0.001	0.038 [0.035; 0.041]	0.981	0.976	0.029
Loading invariance	923.678	384	<0.001	0.036 [0.033; 0.039]	0.982	0.978	0.030
Intercept invariance	985.123	438	<0.001	0.034 [0.031; 0.037]	0.982	0.981	0.034
**Study 3 (Finnish participants,** ***n*** ** = 578)**							
Measurement invariance (six-factor model)							
Configural invariance	848.377	360	<0.001	0.060 [0.055; 0.065]	0.961	0.951	0.047
Loading invariance	895.648	384	<0.001	0.060 [0.055; 0.065]	0.959	0.951	0.055
Intercept invariance	920.304	438	<0.001	0.057 [0.052; 0.062]	0.960	0.958	0.067
**Study 4 (Japanese participants,** ***n*** ** = 228)**							
Measurement invariance (six-factor model)							
Configural invariance	574.054	360	<0.001	0.068 [0.058; 0.078]	0.945	0.930	0.061
Loading invariance	603.652	384	<0.001	0.066 [0.056; 0.076]	0.944	0.934	0.066
Intercept invariance	658.363	438	<0.001	0.063 [0.053; 0.073]	0.945	0.942	0.081

We examined four aspects of invariance in the present study. In the first step we tested configural invariance (Brown, 2006). Standardized factor loadings for the first-order OJC model are presented in Table 3 by time of measurement. All factor loadings were moderate to high and significant (all *p*s < 0.05), ranging from 0.65 to 0.87. Model fit indices showed a good to excellent fit for the configural invariance model (Table 2). In the next step, we tested the loading invariance assumption. Model fit was good to excellent (Table 2). The comparison in model fit between the configural invariance model and loading invariance model was not significant (Δ*χ*^2^ = 25.283, Δ*df* = 24, *p* = 0.391). In a subsequent step, we tested whether the occasion-specific covariance matrices and intercepts were equal across the three waves of measurement. Model fit was good to excellent (Table 2). The results indicated that the constrained model characterized the data excellently and model fit was not significantly different from the configural model (Δ*χ*^2^ = 87.028, Δ*df* = 78, *p* = 0.227). In sum, these results suggest strong measurement invariance for the NOCS.

**Table 3:** Standardized Factor Loadings of the NOCS Items, Study 2.

Item	T1		T2		T3	
	Factor loading	*SE*	Factor loading	*SE*	Factor loading	*SE*
OJC for detachment 1	0.785		0.815		0.850	
OJC for detachment 2	0.767	0.031	0.766	0.032	0.811	0.028
OJC for detachment 3	0.845	0.030	0.863	0.033	0.842	0.032
OJC for relaxation 1	0.842		0.838		0.846	
OJC for relaxation 2	0.772	0.027	0.773	0.035	0.754	0.039
OJC for relaxation 3	0.755	0.027	0.706	0.032	0.742	0.029
OJC for autonomy 1	0.695		0.704		0.730	
OJC for autonomy 2	0.616	0.032	0.624	0.041	0.640	0.046
OJC for autonomy 3	0.736	0.037	0.732	0.045	0.758	0.046
OJC for mastery 1	0.765		0.766		0.780	
OJC for mastery 2	0.662	0.034	0.696	0.040	0.694	0.043
OJC for mastery 3	0.782	0.031	0.779	0.034	0.819	0.040
OJC for meaning 1	0.796		0.741		0.758	
OJC for meaning 2	0.690	0.027	0.668	0.032	0.646	0.039
OJC for meaning 3	0.717	0.028	0.732	0.034	0.703	0.040
OJC for affiliation 1	0.810		0.847		0.838	
OJC for affiliation 2	0.792	0.027	0.806	0.029	0.847	0.030
OJC for affiliation 3	0.861	0.027	0.864	0.030	0.858	0.031

German, Austrian and Swiss participants (*n* = 2,105). The first item within each factor was fixed at 1.00 to establish the scale; hence, there are no standard errors for these six items. All loadings are significant at *p* < 0.001. OJC = off-job crafting. Item numbers are the same as in Study 1 (items in bold face, Table 1).

Test–retest correlations for the OJC dimensions ranged from 0.46 to 0.57 (T1 to T2), 0.49 to 0.63 (T2 to T3), and 0.48 to 0.56 (T1 to T3), respectively. These findings are comparable to the test–retest correlations of 0.50–0.67 found for job crafting in studies with similar time lags between measurements (Lu et al., 2014; Vogt et al., 2016). All test–retest reliabilities of OJC were above 0.40 over a period of three to 6 months, demonstrating both test–retest stability and variability across time (Robinson et al., 1991).

For criterion validity evidence, we first examined how much variance was left unaccounted for after controlling for the baseline (T1) measurement for each outcome in the partial correlation analyses. The unaccounted variance ranged from 37% (work engagement) to 72% (life satisfaction). Thus, over half of the variance for work engagement at T2 and T3 was explained by work engagement at T1. The partial correlations showed that all OJC dimensions were positively related to life satisfaction over time, both after time periods of 3 months and 6 months (Table 4). Thus, Hypothesis 3a was supported. All OJC dimensions except crafting for detachment were positively related to job satisfaction after 3 months, but only crafting for affiliation was positively related to job satisfaction after 6 months. Thus, Hypothesis 3c was partially supported. For work engagement, crafting for detachment was negatively related to work engagement after 3 months, whereas crafting for mastery was positively related to work engagement after 6 months. The other OJC dimensions were not significantly related to work engagement over time. Thus, Hypothesis 3e was partially supported.

**Table 4:** Zero-order (Cross-sectional and Inter-scale) correlations and partial correlations of the OJC dimensions, Study 2.

	*M*	*SD*	OJC for Det T1	OJC for Rel T1	OJC for Aut T1	OJC for Mas T1	OJC for Mea T1	OJC for Aff T1
Age	43.68	11.13	−0.00	0.06^**^	0.02	0.03	0.03	0.02
Gender	1.48	0.50	0.05^*^	0.01	0.01	−0.03	0.05^*^	0.08^*^
OJC for detachment T1	3.86	0.88		0.64^**^	0.46^**^	0.32^**^	0.33^**^	0.35^**^
OJC for relaxation T1	3.73	0.85			0.54^**^	0.41^**^	0.37^**^	0.36^**^
OJC for autonomy T1	3.78	0.75				0.64^**^	0.59^**^	0.47^**^
OJC for mastery T1	3.43	0.82					0.64^**^	0.41^**^
OJC for meaning T1	3.59	0.78						0.60^**^
OJC for affiliation T1	3.80	0.84						
OJC for detachment T2	3.92	0.86	0.56^**^	0.40^**^	0.29^**^	0.20^**^	0.23^**^	0.21^**^
OJC for relaxation T2	3.79	0.82	0.42^**^	0.54^**^	0.37^**^	0.26^**^	0.25^**^	0.22^**^
OJC for autonomy T2	3.83	0.74	0.27^**^	0.33^**^	0.46^**^	0.38^**^	0.35^**^	0.27^**^
OJC for mastery T2	3.43	0.82	0.17^**^	0.22^**^	0.34^**^	0.51^**^	0.42^**^	0.28^**^
OJC for meaning T2	3.61	0.77	0.21^**^	0.22^**^	0.33^**^	0.45^**^	0.52^**^	0.39^**^
OJC for affiliation T2	3.81	0.84	0.24^**^	0.25^**^	0.30^**^	0.31^**^	0.39^**^	0.57^**^
OJC for detachment T3	3.97	0.87	0.51^**^	0.43^**^	0.28^**^	0.18^**^	0.19^**^	0.20^**^
OJC for relaxation T3	3.83	0.83	0.41^**^	0.56^**^	0.37^**^	0.29^**^	0.24^**^	0.20^**^
OJC for autonomy T3	3.86	0.75	0.32^**^	0.39^**^	0.48^**^	0.38^**^	0.37^**^	0.27^**^
OJC for mastery T3	3.47	0.84	0.21^**^	0.29^**^	0.38^**^	0.55^**^	0.44^**^	0.28^**^
OJC for meaning T3	3.62	0.77	0.24^**^	0.25^**^	0.37^**^	0.46^**^	0.56^**^	0.37^**^
OJC for affiliation T3	3.82	0.84	0.29^**^	0.27^**^	0.31^**^	0.32^**^	0.39^**^	0.55^**^
Life satisfaction T1	6.88	2.16	0.13^**^	0.18^**^	0.21^**^	0.16^**^	0.23^**^	0.31^**^
Life satisfaction T2^a^	7.00	2.08	0.12^**^	0.12^**^	0.14^**^	0.15^**^	0.16^**^	0.18^**^
Life satisfaction T3^a^	7.04	2.07	0.07^*^	0.08^**^	0.14^**^	0.08^*^	0.13^**^	0.15^**^
Job satisfaction T1	6.30	2.14	0.07^**^	0.13^**^	0.14^**^	0.14^**^	0.14^**^	0.17^**^
Job satisfaction T2^a^	6.47	2.09	0.05	0.09^**^	0.08^**^	0.08^**^	0.08^**^	0.10^**^
Job satisfaction T3^a^	6.42	2.13	0.01	0.04	0.04	0.03	0.03	0.08^**^
Work engagement T1	3.43	1.10	0.05^*^	0.17^**^	0.21^**^	0.28^**^	0.25^**^	0.26^**^
Work engagement T2^a^	3.43	1.09	−0.05^*^	0.00	0.03	0.04	0.02	0.02
Work engagement T3^a^	3.39	1.12	−0.03	0.02	0.02	0.06^*^	0.03	0.04

German, Austrian and Swiss participants (*n* = 2,105). Abbreviations OJC = off-job crafting, Det = detachment, Rel = relaxation, Aut = autonomy, Mas = mastery, Mea = meaning, Aff = affiliation. Gender coded as 1 = male, 2 = female. Color scheme: darker shades indicate stronger correlations.

* *p* < 0.05;

** *p *< 0.01.

a Partial correlations, controlled for T1 outcome.

## Study 3: Structural, convergent, discriminant, criterion and incremental validity evidence in data with three repeated measurements

In Study 3, we replicated the six-factor solution of the NOCS in a longitudinal confirmatory factor analysis and examined the structural, convergent, discriminant, criterion, and incremental validity evidence of OJC (Hypotheses 2–6) with data collected in Finland.

### Methods

We conducted a study with three measurement points in Finnish organizations. For similar reasons as described in Study 2, we used three-month time lags between each measurement point, with data collection starting in September 2018. Workers had to work a minimum of 24 h per week in order to participate. As an incentive to participate in the study, participants received individualized feedback on their well-being. A convenience sample of workers was recruited through HR personnel in mainly public organizations, including 50–65 year-old workers from a large trade union (*n* = 221), and workers of all ages from cities and municipalities (*n* = 186), other trade union members (*n* = 40), churches (*n* = 19) and an IT company (*n* = 4). Moreover, 38 participants from an earlier study agreed to participate in the study and a further 70 were recruited through social media. In total, 578 workers agreed to participate in the study. Out of the participants, 323 workers (56%) filled all three questionnaires. In addition, three participants’ responses were removed due to failing at all three attention checks used at each measurement point. A majority (85%) of the participants were female. Mean age was 49 (*SD* = 10.23), and half of the participants (50%) had a college or higher-level qualification. Participants worked mainly in health care or social services (37%), public administration (20%), teaching (11%), and the service sector (9%). Participants worked an average of 38.9 h per week (including work hours above contractual hours), ranging from 24 to 65 h. Nonresponse analysis showed no significant differences on age, gender, education, or work hours between the respondents who answered all three questionnaires and respondents who answered only one or two questionnaires.

The 18-item version of the NOCS was used to measure OJC over the past month (Table 1, items in bold face; see Table 5 for the scale intercorrelations in Study 3). Cronbach’s α ranged from 0.89 to 0.92 for crafting for detachment, from 0.85 to 0.89 for crafting for relaxation, from 0.86 to 0.89 for crafting for autonomy, from 0.70 to 0.75 for crafting for mastery, from 0.87 to 0.89 for crafting for meaning, and from 0.86 to 0.90 for crafting for affiliation.

**Table 5:** Intercorrelations of the OJC dimensions, Study 3.

	*M*	*SD*	OJC for Det T1	OJC for Rel T1	OJC for Aut T1	OJC for Mas T1	OJC for Mea T1	OJC for Aff T1
OJC for detachment T1	3.61	1.06		0.58^**^	0.44^**^	0.36^**^	0.42^**^	0.30^**^
OJC for relaxation T1	3.52	0.92			0.64^**^	0.52^**^	0.54^**^	0.44^**^
OJC for autonomy T1	3.77	0.86				0.57^**^	0.64^**^	0.43^**^
OJC for mastery T1	3.10	0.88					0.65^**^	0.45^**^
OJC for meaning T1	3.63	0.95						0.61^**^
OJC for affiliation T1	3.79	0.82						
OJC for detachment T2	3.67	0.96	0.57^**^	0.39^**^	0.36^**^	0.26^**^	0.33^**^	0.28^**^
OJC for relaxation T2	3.52	0.82	0.38^**^	0.56^**^	0.47^**^	0.29^**^	0.34^**^	0.33^**^
OJC for autonomy T2	3.79	0.81	0.20^**^	0.36^**^	0.56^**^	0.30^**^	0.41^**^	0.27^**^
OJC for mastery T2	3.34	0.71	0.23^**^	0.30^**^	0.41^**^	0.55^**^	0.51^**^	0.33^**^
OJC for meaning T2	3.71	0.83	0.23^**^	0.34^**^	0.41^**^	0.41^**^	0.55^**^	0.42^**^
OJC for affiliation T2	3.77	0.83	0.15^**^	0.25^**^	0.23^**^	0.22^**^	0.32^**^	0.61^**^
OJC for detachment T3	3.72	0.94	0.49^**^	0.38^**^	0.33^**^	0.31^**^	0.34^**^	0.28^**^
OJC for relaxation T3	3.58	0.90	0.33^**^	0.52^**^	0.41^**^	0.35^**^	0.43^**^	0.39^**^
OJC for autonomy T3	3.82	0.82	0.22^**^	0.46^**^	0.54^**^	0.40^**^	0.48^**^	0.32^**^
OJC for mastery T3	3.33	0.75	0.23^**^	0.35^**^	0.37^**^	0.58^**^	0.50^**^	0.34^**^
OJC for meaning T3	3.70	0.81	0.22^**^	0.34^**^	0.38^**^	0.44^**^	0.54^**^	0.41^**^
OJC for affiliation T3	3.79	0.84	0.23^**^	0.31^**^	0.27^**^	0.36^**^	0.42^**^	0.56^**^

Finnish participants (*n* = 578). Abbreviations OJC = off-job crafting, Det = detachment, Rel = relaxation, Aut = autonomy, Mas = mastery, Mea = meaning, Aff = affiliation. Color scheme: darker shades indicate stronger correlations.

** *p* < 0.01.

Proactive personality was measured at T1 with a six-item version of the Proactive Personality Scale (Bateman and Crant, 1993; Claes et al., 2005). An example item is “I excel at identifying opportunities.” The scale for proactive personality ranged from 1 (“totally disagree”) to 5 (“totally agree”). Cronbach’s α was 0.80. Leisure crafting was measured at T1 with the scale by Petrou and Bakker (2016). An example item is “Over the past month, I tried to build relationships through leisure activities.” The scale ranged from 1 (“never”) to 5 (“very often”). Cronbach’s α was 0.92. Recreational activities were measured at T1 with individual items adapted from earlier studies (Tinsley and Eldredge, 1995; Demerouti et al., 2009; Brajša-Žganec et al., 2011) and activities mentioned in qualitative interviews in the scale development phase. All items started with “During off-job time over the past month, I have engaged in ….” The items were “Resting (e.g., napping, listening to music, reading, watching tv),” “Relaxation activities (e.g., yoga, massage, sauna),” “Volunteering (e.g., organizing events, serving the community, medical volunteering),” “Creative activities (e.g., playing a musical instrument, arts or crafts),” “Reflection (e.g., thinking about the past and the future, writing a diary, storytelling)” and “Active socializing (e.g., going out with friends, visiting friends or relatives).” Response options ranged from 1 = “not at all or once a month” to 6 = “several times a day.”

DRAMMA needs satisfaction was measured at all-time points with previously validated measures with three items per dimension. All items started with “Over the past month, …” and answering scales ranged from 1 (“Not agree at all”) to 5 (“Fully agree): Following Kujanpää et al. (2020) and Kuykendall et al. (2017), detachment and relaxation were measured with the recovery experience questionnaire by Sonnentag and Fritz (2007). Example items are “During time after work, I forgot about work” for detachment, and “I kicked back and relaxed.” Autonomy, competence (mastery) and relatedness (affiliation) were measured with the basic psychological need satisfaction scale by Chen et al. (2015). Example items are “I’ve felt my choices expressed who I really am” for autonomy, “I’ve felt capable at what I did” for competence, and “I’ve felt connected with people who care for me, and for whom I care” for relatedness. Meaning was measured with the Meaning in Life Questionnaire (Steger et al., 2006). An example item is “At this point of time in my life, my life has a clear sense of purpose.” Cronbach’s α ranged from 0.83 to 0.91.

Life satisfaction and job satisfaction were measured at all-time points with single-item measures adapted from Van den Broeck et al. (2010). The items were “How satisfied have you been with your private life over the past month?” for life satisfaction, and “How satisfied have you been with your job over the past month?” for job satisfaction. The answering scale for these items ranged from 1 (“very dissatisfied”) to 10 (“very satisfied”). Task- and relationship-level family role performance were measured at all-time points with a scale developed and validated by Chen et al. (2014). Each item was presented with the question “To what extent do you think you fulfilled what was expected of you in relation to the following aspects of your current family life over the past month?.” An example item is “Completing household responsibilities.” The answering scale ranged from 1 (“did not fulfill expectations at all”) to 5 (“fulfilled expectations completely”). Cronbach’s α ranged from 0.87 to 0.89. Perceived current work ability compared to lifetime best was measured at all-time points with a single item “How many points would you give your current ability to work?” from the Work Ability Index (WAI), which has been shown to accurately reflect the total WAI measure (Ilmarinen, 2006; McGonagle et al., 2015; Jääskeläinen et al., 2016). The answering scale ranged from 0 (“cannot currently work at all”) to 10 (“work ability at its lifetime best”). Work engagement was measured at all-time points as in Study 2 (Schaufeli et al., 2006, vigor and dedication dimensions combined), with the exception that a slightly different response scale ranging from 1 (“never”) to 6 (“daily”) was used. Cronbach’s α were 0.94 at all-time points for work engagement.

The Harman single-factor test showed that the obtained single factor accounted for 21.26% of the total variance, indicating that common method bias was not present.

### Results

First, we examined the fit of the six-factor model of the NOCS among Finnish participants. The model fit was between excellent and good (*χ*^2^ = 824.504, *df* = 360, *p* < 0.001, R-RMSEA = 0.062 (90%CI [0.056; 0.067]), R-CFI = 0.961, R-TLI = 0.950, SRMR = 0.048). Model fit indices showed a good to acceptable fit for the configural invariance model and the loading invariance model (Table 2). The comparison in model fit between the configural invariance model and loading invariance model was significant (Δ*χ*^2^ = 47.271, Δ*df* = 24, *p* = 0.003). Fit for the intercept invariance model varied from good to acceptable (Table 2). The results indicated that the constrained model characterized the data well and model fit was not significantly different from the configural model (Δ*χ*^2^ = 71.927, Δ*df* = 78, *p* = 0.672). In sum, similar to Study 2, these results suggest strong measurement invariance for the NOCS.

Of the OJC dimensions, proactive personality was positively related to OJC for autonomy, for mastery, for meaning, and for affiliation (*r*’s ranging from 0.13 to 0.26, *p* < 0.01, Table 6), whereas proactive personality was unrelated to OJC for detachment (*r* = −0.05, *p* > 0.05) and for relaxation (*r* = 0.06, *p* > 0.05). Thus, Hypothesis 4b received partial support. Moreover, all OJC dimensions correlated positively with the existing scale on leisure crafting (Petrou and Bakker, 2016; *r*’s 0.15–0.57, *p* < 0.01). Thus, Hypothesis 4c was supported. The correlation between crafting for mastery and leisure crafting was highest, indicating that this OJC dimension is most similar to leisure crafting.

**Table 6:** Zero-order (cross-sectional) correlations and partial correlations of the OJC dimensions, Study 3.

	*M*	*SD*	OJC for Det T1	OJC for Rel T1	OJC for Aut T1	OJC for Mas T1	OJC for Mea T1	OJC for Aff T1
Age	48.70	10.23	0.01	0.13^**^	0.15^**^	0.10^*^	0.10^*^	0.07
Gender	1.86	0.36	0.10^*^	0.06	0.05	−0.05	0.05	0.02
Proactive personality T1	3.69	0.68	−0.05	0.06	0.13^**^	0.26^**^	0.16^**^	0.17^**^
Leisure crafting T1	2.56	0.82	0.15^**^	0.23^**^	0.26^**^	0.57^**^	0.41^**^	0.30^**^
Resting T1	4.66	0.99	0.13^**^	0.26^**^	0.18^**^	0.08	0.09^*^	0.10^*^
Relaxation activities T1	2.66	1.30	0.09^*^	0.16^**^	0.14^**^	0.13^**^	0.17^**^	0.19^**^
Volunteering activities T1	1.76	1.15	0.01	0.02	0.08	0.24^**^	0.21^**^	0.14^**^
Creative activities T1	2.08	1.49	0.02	0.05	0.06	0.24^**^	0.08	0.08
Reflection T1	2.93	1.63	0.14^**^	0.17^**^	0.11^*^	0.19^**^	0.16^**^	0.12^**^
Active socializing T1	2.75	1.17	0.13^**^	0.15^**^	0.20^**^	0.22^**^	0.20^**^	0.27^**^
Detachment T1	3.18	1.10	0.34^**^	0.31^**^	0.31^**^	0.18^**^	0.25^**^	0.19^**^
Detachment T2^a^	3.19	1.08	−0.00	0.10	0.14^**^	0.16^**^	0.10	0.11^*^
Detachment T3^a^	3.20	1.04	0.03	0.13^*^	0.13^**^	0.06	0.04	0.09
Relaxation T1	3.70	0.90	0.32^**^	0.54^**^	0.48^**^	0.24^**^	0.32^**^	0.29^**^
Relaxation T2^a^	3.66	0.95	0.15^**^	0.13^*^	0.26^**^	0.13^*^	0.13^*^	0.11^*^
Relaxation T3^a^	3.77	0.88	0.14^**^	0.22^**^	0.21^**^	0.14^*^	0.13^*^	0.11^*^
Autonomy T1	3.60	0.76	0.06	0.28^**^	0.32^**^	0.35^**^	0.34^**^	0.35^**^
Autonomy T2^a^	3.60	0.76	0.01	0.10	0.10	0.13^*^	0.10^*^	0.14^**^
Autonomy T3^a^	3.60	0.79	−0.01	0.10	0.15^**^	0.14^*^	0.21^**^	0.25^**^
Competence T1	3.97	0.68	0.00	0.19^**^	0.23^**^	0.28^**^	0.24^**^	0.30^**^
Competence T2^a^	3.94	0.71	−0.06	0.12^*^	0.11^*^	0.07	0.15^**^	0.14^**^
Competence T3^a^	4.01	0.67	0.02	0.02	0.12^*^	0.19^**^	0.13^*^	0.17^**^
Meaning T1	3.86	0.79	0.06	0.25^**^	0.33^**^	0.33^**^	0.43^**^	0.48^**^
Meaning T2^a^	3.82	0.83	0.02	0.06	−0.01	0.03	0.12^*^	0.07
Meaning T3^a^	3.86	0.80	0.08	0.14^**^	0.14^**^	0.13^*^	0.23^**^	0.20^**^
Relatedness T1	4.26	0.69	0.02	0.16^**^	0.19^**^	0.15^**^	0.24^**^	0.46^**^
Relatedness T2^a^	4.25	0.65	0.04	0.08	0.08	0.08	0.09	0.18^**^
Relatedness T3^a^	4.26	0.65	0.10	0.16^**^	0.11^*^	0.14^**^	0.19^**^	0.13^*^
Life satisfaction T1	7.58	1.83	0.16^**^	0.33^**^	0.40^**^	0.23^**^	0.39^**^	0.48^**^
Life satisfaction T2^a^	7.62	1.76	−0.01	0.01	0.07	0.06	0.06	0.04
Life satisfaction T3^a^	7.66	1.70	0.11^*^	0.09	0.05	0.07	0.13^*^	0.10
Family task performance T1	3.83	0.85	0.10^*^	0.16^**^	0.29^**^	0.17^**^	0.20^**^	0.23^**^
Family task performance T2^a^	3.91	0.80	0.01	0.03	0.11^*^	0.10	0.11^*^	0.11^*^
Family task performance T3^a^	3.98	0.77	0.01	0.14^*^	0.12^*^	0.15^**^	0.16^**^	0.12^*^
Family relat. Performance T1	3.78	0.90	0.11^*^	0.19^**^	0.24^**^	0.26^**^	0.33^**^	0.41^**^
Family relat. Performance T2^a^	3.78	0.85	0.03	0.01	0.02	−0.01	0.02	0.14^**^
Family relat. Performance T3^a^	3.80	0.86	0.00	−0.02	−0.03	0.06	0.02	0.07
Job satisfaction T1	7.26	2.05	−0.10^*^	0.10^*^	0.15^**^	0.16^**^	0.19^**^	0.24^**^
Job satisfaction T2^a^	7.37	1.90	−0.07	0.04	0.03	0.08	0.06	0.05
Job satisfaction T3^a^	7.29	2.01	−0.06	0.01	0.05	0.08	0.08	0.06
Perceived work ability T1	7.79	1.65	−0.06	0.12^**^	0.21^**^	0.19^**^	0.23^**^	0.21^**^
Perceived work ability T2^a^	7.72	1.58	0.05	0.14^**^	0.14^**^	0.16^**^	0.18^**^	0.14^**^
Perceived work ability T3^a^	7.74	1.72	0.04	0.07	0.13^*^	0.06	0.08	0.04
Work engagement T1	4.59	1.22	−0.05	0.15^**^	0.19^**^	0.26^**^	0.24^**^	0.34^**^
Work engagement T2^a^	4.54	1.21	−0.12^*^	−0.01	−0.04	−0.07	−0.02	0.04
Work engagement T3^a^	4.58	1.18	−0.01	0.09	0.09	0.09	0.11^*^	0.15^**^

Finnish participants (*n* = 578). Abbreviations OJC = off-job crafting, Det = detachment, Rel = relaxation, Aut = autonomy, Mas = mastery, Mea = meaning, Aff = affiliation, relat. Performance = relationship performance. Gender coded as 1 = male, 2 = female. Color scheme: darker shades indicate stronger correlations.

* *p* < 0.05;

** *p *< 0.01.

a Partial correlations, controlled for T1 outcome.

To test Hypothesis 6, we examined a six-factor model in confirmatory factor analysis with one recreational activity added to each OJC dimension at T1. Based on earlier literature (e.g., Gagné, 2003; Zijlstra and Sonnentag, 2006; Van Tilburg et al., 2013; Tuisku et al., 2016), we paired each activity item with its corresponding three crafting items (resting with the dimension of OJC for detachment, relaxation activities with OJC for relaxation, volunteering activities with OJC for autonomy, creative activities with OJC for mastery, reflection with OJC for meaning, and active socializing with OJC for affiliation). If recreational activities can be distinguished from OJC, the activity items would be expected to show a poor loading (<0.30, Brown, 2006) on their designated factor in the six-factor solution. The zero-order correlations between OJC dimensions and corresponding recreational activities were rather low, ranging from 0.08 to 0.27. The overall model fit was acceptable [*χ*^2^(237) = 792.18, *p* > 0.001, CFI = 0.91, TLI = 0.89, RMSEA = 0.06]. Standardized loadings for the crafting items ranged from 0.63 to 0.95 on each factor, whereas the activity items loaded only weakly on each of their designated factors (standardized loadings ranged from 0.11 to 0.28), indicating non-salient loadings (<0.30) for the activity items (Brown, 2006). Thus, Hypothesis 6 was supported. OJC dimensions could be clearly distinguished from each designated recreational activity.

Prior to testing Hypothesis 2, we examined whether OJC is distinct from needs satisfaction (detachment, relaxation, autonomy, competence, meaning, and relatedness) at T1 with Average Variance Extracted estimates (Fornell and Larcker, 1981; Farrell, 2010). The correlations between OJC dimensions and needs satisfaction were mostly significant (*r*’s 0.00–0.54). The AVE estimates for needs satisfaction ranged from 0.62 to 0.76, and for OJC from 0.48 to 0.79, whereas the squared factor-level correlations between needs satisfaction and OJC ranged from 0.00 to 0.39, demonstrating that OJC is a distinct construct from needs satisfaction, but related (as predicted by our theoretical model in which needs satisfaction constitutes a key outcome of crafting). In the partial correlations from OJC at T1 and needs satisfaction at T2 and T3, the variance left unaccounted for after controlling for the baseline needs satisfaction ranged from 53% (detachment) to 77% (autonomy). OJC for detachment was not related to experienced detachment (Table 6). On the other hand, OJC for relaxation was positively related to experienced relaxation after 3 months and after 6 months. OJC for autonomy was not related to experienced autonomy after 3 months but was positively related to autonomy after 6 months. Similarly, OJC for mastery was not related to experienced competence after 3 months but was positively related to competence after 6 months. OJC for meaning was positively related to experienced meaning both after three and after 6 months. Similarly, OJC for affiliation was positively related to experienced relatedness both after three and after 6 months. The zero-order correlations (without controlling for baseline) between OJC at T1 and needs satisfaction at T2 and T3 were all positive and significant (*r*’s 0.21–0.49, *p* < 0.01). To summarize, Hypothesis 2 received partial support.

Regarding criterion validity evidence, the variance left unaccounted for after controlling for the baseline in each outcome ranged from 46% (work engagement) to 74% (life satisfaction). OJC was not related to life satisfaction after 3 months, whereas the OJC dimensions of crafting for detachment and for meaning were positively related to life satisfaction after 6 months (Table 6). Thus, Hypothesis 3a received partial support. Crafting for meaning and for affiliation were positively related to family role task performance after 3 months and all OJC dimensions except crafting for detachment were positively related to family role task performance after 6 months. Only crafting for affiliation was positively related to family role relational performance after 3 months. OJC was not related to family role relational performance after 6 months. Thus, Hypothesis 3b received support for family role task performance, whereas only crafting for affiliation was related to the relational dimension of family role performance. OJC was not related to job satisfaction, either after 3 months or after 6 months. Thus, Hypothesis 3c was not supported. All OJC dimensions except crafting for detachment were positively related to perceived work ability after 3 months, whereas only crafting for autonomy was positively related to work ability after 6 months. Thus, Hypothesis 3d received partial support. Crafting for detachment was negatively related to work engagement after 3 months, whereas crafting for meaning and for affiliation were positively related to work engagement after 6 months. Thus, Hypothesis 3e received partial support.

Next, we tested the incremental validity evidence of OJC with hierarchical multiple regression analyses at T1. When proactive personality was entered as a predictor for optimal functioning in the first step, and the OJC dimensions were entered in the second step, OJC explained significant variance beyond proactive personality for all outcomes, i.e., life satisfaction (step 1: R^2^ = 0.01, step 2: ΔR^2^ = 0.27, *p* change <0.001), family role task performance (step 1: R^2^ = 0.00, step 2: ΔR^2^ = 0.10, *p* change <0.001), family role relational performance (step 1: R^2^ = 0.02, step 2: ΔR^2^ = 0.16, *p* change <0.001), job satisfaction (step 1: R^2^ = 0.04, step 2: ΔR^2^ = 0.08, *p* change <0.001), work ability (step 1: R^2^ = 0.03, step 2: ΔR^2^ = 0.09, *p* change <0.001), and work engagement (step 1: R^2^ = 0.06, step 2: ΔR^2^ = 0.13, *p* change <0.001). Similarly, OJC explained significant variance beyond leisure crafting for all outcomes, i.e., life satisfaction (step 1: R^2^ = 0.07, step 2: ΔR^2^ = 0.23, *p* change <0.001), family role task performance (step 1: R^2^ = 0.02, step 2: ΔR^2^ = 0.09, *p* change <0.001), family role relational performance (step 1: R^2^ = 0.05, step 2: ΔR^2^ = 0.15, *p* change <0.001), job satisfaction (step 1: R^2^ = 0.04, step 2: ΔR^2^ = 0.08, *p* change <0.001), work ability (step 1: R^2^ = 0.05, step 2: ΔR^2^ = 0.08, *p* change <0.001), and work engagement (step 1: R^2^ = 0.07, step 2: ΔR^2^ = 0.11, *p* change <0.001). Thus, Hypotheses 5b and 5c received support, since OJC predicted variance in all optimal functioning outcomes beyond proactive personality and leisure crafting.

## Study 4: Structural and criterion validity evidence in data with three repeated measurements

In Study 4, we examined the structural and criterion validity evidence of OJC (Hypothesis 3) with data collected in Japan, a non-Western country.

### Methods

We conducted a study with three measurement points in Japanese organizations. As in Studies 2 and 3, we used three-month time lags between measurement points, with data collection starting in December 2018. Participants were recruited with convenience sampling through a consultancy agency with established contacts in various Japanese companies. Workers had to work a minimum of 24 h per week in order to participate. As an incentive to participate in the study, participants received individualized feedback on their well-being. In total, 228 workers agreed to participate in the study. A total of 115 workers (50% of all participants) filled all three questionnaires. None of the participants failed at all three attention checks used at each measurement point, and thus all answers were retained. A slight majority (64%) of the participants were male. Mean age was 31 (*SD* = 6.35), and 95% had a college or higher-level qualification. Participants worked mainly in information technology (57%), but also in various other fields such as health care. Participants worked an average of 48.3 h per week (including work hours above contractual hours), ranging from 24 to 80. Nonresponse analysis showed no significant differences on age, gender, education, or work hours between the respondents who answered all three questionnaires and respondents who answered only one or two questionnaires.

The 18-item version of the NOCS was used to measure OJC over the past month at all-time points (Table 1, items in bold face). Cronbach’s α ranged from 0.77 (OJC for detachment) to 0.90 (OJC for affiliation).

Life satisfaction and job satisfaction were measured at all-time points with single items as in Study 3 (Van den Broeck et al., 2010), with the exception that due to a coding error, life satisfaction was measured on a scale of 1 (“very dissatisfied”) to 5 (“very satisfied”) at T1. For better comparability, the values at T1 were linearly transformed to a 1 to 10 scale. Task- and relationship-level family role performance was measured at all-time points as in Study 3 (Chen et al., 2014). Cronbach’s α ranged from 0.88 to 0.92 for family task performance, and from 0.90 to 0.93 for family relationship performance. Work ability was measured at all-time points with a single item as in Study 3 (Ilmarinen, 2006; McGonagle et al., 2015). Work engagement was measured at all-time points as in Studies 2 and 3 (Schaufeli et al., 2006, vigor and dedication dimensions combined). Cronbach’s α ranged from 0.92 to 0.95 for work engagement.

The Harman single-factor test showed that the obtained single factor accounted for 18.00% of the total variance, indicating that common method bias was not present.

### Results

First, we examined the fit of the six-factor model of the NOCS among Japanese participants. The model fit was between good and acceptable (*χ*^2^ = 574.667, *df* = 360, *p* < 0.001, R-RMSEA = 0.070 (90%CI [0.059; 0.080]), R-CFI = 0.945, R-TLI = 0.930, SRMR = 0.061). Fits for the configural invariance and the loading invariance model were acceptable (Table 2). The difference in model fit between the configural invariance model and loading invariance model was not significant (Δ*χ*^2^ = 29.598, Δ*df* = 24, *p* = 0.198). Fit for the intercept invariance model was good to acceptable (Table 2). Results indicated that the constrained model characterized the data well and model fit was not significantly different from the configural model (Δ*χ*^2^ = 84.309, Δ*df* = 78, *p* = 0.293). Thus, similar to Studies 2 and 3, the results suggested strong measurement invariance. For all crafting dimensions, Japanese participants crafted their off-job lives less than the participants in the US, German-speaking countries, Finland, or the United Kingdom (see means in Tables 1, 4, 5, 7, and 8).

**Table 7:** Zero-order (cross-sectional and inter-scale) correlations and partial correlations of the OJC dimensions, Study 4.

	*M*	*SD*	OJC for Det T1	OJC for Rel T1	OJC for Aut T1	OJC for Mas T1	OJC for Mea T1	OJC for Aff T1
Age	30.86	6.35	−0.06	−0.16^*^	−0.12	−0.14^*^	−0.20^**^	−0.06
Gender	1.37	0.50	0.05	0.18^**^	0.11	−0.03	0.07	0.23^**^
OJC for detachment T1	2.77	1.04		0.67^**^	0.63^**^	0.24^**^	0.34^**^	0.44^**^
OJC for relaxation T1	3.14	1.04			0.76^**^	0.33^**^	0.42^**^	0.56^**^
OJC for autonomy T1	2.82	1.10				0.48^**^	0.61^**^	0.55^**^
OJC for mastery T1	3.07	1.02					0.64^**^	0.34^**^
OJC for meaning T1	2.55	1.16						0.53^**^
OJC for affiliation T1	2.82	1.01						
OJC for detachment T2	2.78	1.03	0.49^**^	0.43^**^	0.36^**^	0.26^**^	0.22^**^	0.40^**^
OJC for relaxation T2	3.04	1.01	0.44^**^	0.57^**^	0.47^**^	0.25^**^	0.32^**^	0.45^**^
OJC for autonomy T2	2.84	1.08	0.42^**^	0.52^**^	0.50^**^	0.33^**^	0.38^**^	0.37^**^
OJC for mastery T2	2.97	0.94	0.25^**^	0.25^**^	0.33^**^	0.47^**^	0.35^**^	0.30^**^
OJC for meaning T2	2.68	1.07	0.17	0.24^**^	0.33^**^	0.44^**^	0.57^**^	0.31^**^
OJC for affiliation T2	2.80	1.08	0.30^**^	0.35^**^	0.29^**^	0.19^*^	0.33^**^	0.63^**^
OJC for detachment T3	3.11	0.94	0.41^**^	0.40^**^	0.34^**^	0.21^**^	0.12	0.10
OJC for relaxation T3	3.25	0.99	0.36^**^	0.49^**^	0.36^**^	0.30^**^	0.19^*^	0.23^**^
OJC for autonomy T3	3.08	1.06	0.42^**^	0.47^**^	0.45^**^	0.28^**^	0.30^**^	0.28^**^
OJC for mastery T3	2.92	0.90	0.11	0.18	0.28^**^	0.51^**^	0.34^**^	0.10
OJC for meaning T3	2.73	1.02	0.29^**^	0.27^**^	0.37^**^	0.45^**^	0.47^**^	0.24^**^
OJC for affiliation T3	2.78	0.98	0.22^*^	0.31^**^	0.24^**^	0.14	0.21^*^	0.43^**^
Life satisfaction T1	7.21	2.09	0.15^*^	0.24^**^	0.30^**^	0.25^**^	0.27^**^	0.32^**^
Life satisfaction T2^a^	7.02	2.01	0.13	0.02	0.06	0.16	0.19^*^	0.05
Life satisfaction T3^a^	7.05	1.91	0.14	0.09	0.06	0.10	0.01	0.14
Family task performance T1	3.28	0.99	0.18^*^	0.13	0.17^*^	0.20^**^	0.20^**^	0.14
Family task performance T2^a^	3.28	1.06	0.15	0.13	0.06	0.06	0.19^*^	0.15
Family task performance T3^a^	3.25	0.98	0.08	0.09	0.11	0.02	0.11	0.14
Family relat. Performance T1	2.96	1.12	0.19^**^	0.11	0.14	0.09	0.17^*^	0.19^**^
Family relat. Performance T2^a^	2.92	1.21	0.11	0.15	0.11	0.14	0.18	0.19^*^
Family relat. Performance T3^a^	2.79	1.11	0.07	0.12	0.08	−0.08	0.04	0.05
Job satisfaction T1	6.44	2.36	−0.06	−0.11	0.04	0.14^*^	0.11	0.05
Job satisfaction T2^a^	6.22	2.13	−0.11	−0.09	−0.06	0.02	0.11	0.04
Job satisfaction T3^a^	5.62	2.22	0.06	−0.03	−0.07	−0.17	−0.06	0.16
Perceived work ability T1	5.54	2.00	0.01	−0.09	0.09	0.04	0.13	−0.02
Perceived work ability T2^a^	5.52	2.10	0.01	0.02	0.04	0.05	0.05	−0.02
Perceived work ability T3^a^	5.81	1.91	0.05	0.09	0.08	−0.00	0.03	0.19^*^
Work engagement T1	4.07	1.30	−0.17^*^	−0.14	−0.08	0.12	0.03	0.01
Work engagement T2^a^	4.13	1.37	−0.12	−0.10	−0.06	−0.08	0.04	−0.01
Work engagement T3^a^	4.10	1.29	0.06	−0.00	0.06	0.03	0.01	0.16

Japanese participants (*n* = 228). Abbreviations OJC = off-job crafting, Det = detachment, Rel = relaxation, Aut = autonomy, Mas = mastery, Mea = meaning, Aff = affiliation, relat. Performance = relationship performance. Gender coded as 1 = male, 2 = female. Color scheme: darker shades indicate stronger correlations.

* *p* < 0.05;

** *p *< 0.01.

a Partial correlations, controlled for T1 outcome.

**Table 8:** Correlations of the OJC dimensions, job crafting and home crafting, Study 5.

	*M*	*SD*	OJC for Det	OJC for Rel	OJC for Aut	OJC for Mas	OJC for Mea	OJC for Aff
Age	31.08	7.19	−0.03	−0.05	−0.01	−0.05	−0.08	0.05
Gender	1.48	0.52	−0.10	−0.05	−0.07	−0.06	0.02	0.08
OJC for detachment	3.83	0.86		0.58^**^	0.57^**^	0.36^**^	0.36^**^	0.31^**^
OJC for relaxation	3.65	0.84			0.70^**^	0.51^**^	0.51^**^	0.35^**^
OJC for autonomy	3.62	0.81				0.67^**^	0.66^**^	0.39^**^
OJC for mastery	3.37	0.82					0.64^**^	0.21^**^
OJC for meaning	3.49	0.83						0.34^**^
OJC for affiliation	3.53	0.90						
Increasing structural JR	3.65	0.73	−0.04	0.16^*^	0.22^**^	0.38^**^	0.34^**^	0.24^**^
Decreasing hindering JD	3.03	0.78	0.21^**^	0.27^**^	0.34^**^	0.21^**^	0.26^**^	0.09
Increasing social JR	2.62	0.91	−0.02	0.08	0.07	0.15^*^	0.16^**^	0.21^**^
Increasing challenging JD	2.94	0.93	−0.13^*^	0.09	0.08	0.28^**^	0.22^**^	0.20^**^
HC seeking resources	3.32	0.57	0.13^*^	0.25^**^	0.30^**^	0.42^**^	0.45^**^	0.32^**^
HC seeking challenges	3.01	0.84	−0.12	0.00	0.17^**^	0.36^**^	0.20^**^	0.19^**^
HC reducing demands	3.15	0.78	0.10	0.22^**^	0.11	0.05	0.09	0.09

UK participants (*n* = 237). Abbreviations OJC = off-job crafting, Det = detachment, Rel = relaxation, Aut = autonomy, Mas = mastery, Mea = meaning, Aff = affiliation, JR = job resources, JD = job demands, HC = home crafting. Gender coded as 1 = male, 2 = female. Color scheme: darker shades indicate stronger correlations.

* *p *< 0.05;

** *p* < 0.01.

For criterion validity evidence, the variance left unaccounted for after controlling for the baseline in each outcome ranged from 44% (family role relational performance) to 86% (life satisfaction). Only OJC for meaning was positively related to life satisfaction after 3 months, but not after 6 months (Table 7). Thus, Hypothesis 3a received partial support. OJC for meaning was positively related to family role task performance after 3 months, but not after 6 months. OJC for affiliation was positively related to family role relational performance after 3 months, but not after 6 months. Thus, Hypothesis 3b received partial support. On the other hand, OJC was not significantly related to job satisfaction over time. Thus, Hypothesis 3c was not supported. OJC for affiliation was positively related to work ability only after 6 months, but not related to work ability after 3 months. Thus, Hypothesis 3d received partial support. OJC was not significantly related to work engagement over time. Thus, Hypothesis 3e was not supported.

## Study 5: Convergent and incremental validity evidence compared to job crafting and home crafting

In Study 5, we examined the convergent and incremental validity evidence of OJC in relation to job crafting (job demands- and resources based; e.g., Tims and Bakker, 2010) and a recently developed scale on home crafting (Demerouti et al., 2020).

### Methods

We recruited a sample of 237 workers *via* Prolific in the United Kingdom. Data were collected in July 2021. Workers were required to work at least 21 h per week to be able to participate. The participants were paid £1.50 for completing the study. None of the participants failed at both two attention checks used, and thus all answers were retained. The participants’ ages varied between 19 and 57 years (mean = 31, *SD* = 7.2) and average weekly working time was 40 h (*SD* = 6.5; range 24–72). Sixty-eight percent of the sample had a bachelor’s degree or higher, and the majority (93%) can be classified as white-collar workers from a variety of sectors. Half of the sample (46%) was female.

Off-job crafting was assessed with the 18-item NOCS (Table 1, items in bold face). Cronbach’s α varied between 0.81 and 0.90 for the subscales. Job crafting was assessed with a validated 21-item job crafting scale (Tims et al., 2012) assessing participants’ attempts to increase structural job resources, decrease hindering job demands, increase social job resources, and increase challenging job demands. Cronbach’s α varied between 0.83 and 0.88 for the subscales. Home crafting was measured with a 12-item scale to measure “seeking for resources,” “seeking challenges,” and “reducing demands” (Demerouti et al., 2020). Cronbach’s α varied between 0.55 and 0.76 for the subscales. We used the same measures for optimal functioning outcomes as in Studies 2 to 4 (life satisfaction, family role performance, job satisfaction, perceived work ability, and work engagement) and Cronbach’s α varied between 0.86 (family role relational performance) and 0.93 (work engagement).

The Harman single-factor test showed that the obtained single factor accounted for 19.31% of the total variance, indicating that common method bias was not present.

### Results

OJC was mostly positively related to job crafting (mean *r* = 0.17), except for one negative correlation between OJC for detachment and “increasing challenging demands” (Table 8). OJC for mastery and for meaning were positively related to all job crafting dimensions. Thus, Hypothesis 4a gained partial support. All OJC dimensions were positively related to home crafting “increasing resources,” whereas only OJC for detachment and for relaxation were related to “decreasing demands.” OJC for autonomy, for mastery, for meaning, and for affiliation were related to “increasing challenges.” Thus, Hypothesis 4d was partially supported.

Correlations further showed that job crafting explained more variance in work-related well-being and less variance in life satisfaction and family role performance than did OJC. “Decreasing hindering demands” did not explain any variance in work- or non-work-related outcomes. Particularly OJC for mastery, for meaning and for affiliation were strongly linked to optimal functioning in both job and off-job domains (*r* ranged between 0.16 and 0.48). Mean *r* across all outcomes was 0.26 for both JC and OJC.

OJC explained variance beyond job crafting in life satisfaction (step 1: R^2^ = 0.07, step 2: ΔR^2^ = 0.24, *p* change <0.001), family role task (step 1: R^2^ = 0.06, step 2: ΔR^2^ = 0.11, *p* change <0.001) and relational performance (step 1: R^2^ = 0.07, step 2: ΔR^2^ = 0.13, *p* change <0.001), and perceived work ability (step 1: R^2^ = 0.13, step 2: ΔR^2^ = 0.05, *p* change <0.05), but not for job satisfaction (step 1: R^2^ = 0.29, step 2: ΔR^2^ = 0.01, *p* change = 0.76) and work engagement (step 1: R^2^ = 0.42, step 2: ΔR^2^ = 0.02, *p* change = 0.31). Thus, Hypothesis 5a was partially supported. Summing up, OJC showed evidence for incremental validity beyond job crafting for predicting optimal functioning in the off-job domain and work ability.

For home crafting, the dimension “reducing demands” did not correlate with any of the outcomes measured. Correlations between home crafting dimensions and optimal functioning, and OJC dimensions and optimal functioning were highly comparable, with an average *r* of 0.23 for OJC and of 0.16 for home crafting.

OJC explained variance beyond home crafting in life satisfaction (step 1: R^2^ = 0.10, step 2: ΔR^2^ = 0.21, *p* change <0.001), and family role task (step 1: R^2^ = 0.12, step 2: ΔR^2^ = 0.08, *p* change <0.01) and relational performance (step 1: R^2^ = 0.10, step 2: ΔR^2^ = 0.11, *p* change <0.001), marginally for perceived work ability (step 1: R^2^ = 0.05, step 2: ΔR^2^ = 0.05, *p* change = 0.08) and work engagement (step 1: R^2^ = 0.14, step 2: ΔR^2^ = 0.05, *p* change = 0.05), and not for job satisfaction (step 1: R^2^ = 0.09, step 2: ΔR^2^ = 0.03, *p* change = 0.39). Thus, Hypothesis 5d gained partial support. Examining the results per dimension, it seems that home crafting does not capture the dimensions of OJC for mastery and affiliation, as this is where the NOCS contributed most. Summing up, home crafting subscales showed low internal consistency and particularly the value of the subscale “reducing demands” was questionable. The explanatory power of both OJC and the home crafting scale were comparable for optimal functioning at work and OJC had additional value in predicting optimal functioning in the off-job domain, demonstrating incremental validity evidence.

## General discussion

In this series of studies, we examined the concept of needs-based OJC and its effects on optimal functioning in two life domains (working life and off-job life) and in five sub-studies with participants from seven countries (the United States, Germany, Austria, Switzerland, Finland, Japan, and the United Kingdom). Please see Appendix 2 for a list of all the hypotheses and results across the different sub-studies.

### Structural validity evidence

In Study 1a, we developed the NOCS and tested the scale in an exploratory factor analysis. Support was found for the EFA six-factor structure and reliability of the NOCS. The structural validity evidence of the six-factor solution was further tested in studies 2–4 with repeated-measurements data. The results showed that the six-factor structure fitted the data better than the other factor structures examined. The results from three sub-studies (Studies 2–4) showed strong measurement invariance for the NOCS across a period of 6 months in both Western (German-speaking countries, Finland) and non-Western countries (Japan), providing support for the internal and test–retest reliability of the scale across different countries and working contexts. To summarize, the NOCS is a reliable instrument which can be applied in several different languages and countries.

### Criterion validity evidence

Hypothesis 1 received support (Study 1b), as the number of crafting activities a person reported was related to their crafting score on the respective OJC dimension. Moreover, participants gave a wide variety of relevant examples for each crafting dimension, demonstrating that the concept of OJC was not only readily comprehensible but also practically applicable in workers’ daily lives.

Hypothesis 2 received partial support (Study 3). OJC for relaxation, for meaning and for affiliation were related to their matching needs satisfaction both after three and after 6 months. For autonomy and competence, the results suggest that OJC may operate on a slower time frame. This means that OJC for autonomy and mastery could produce increased satisfaction for the needs of autonomy and competence slower than within 3 months. On the other hand, crafting for detachment was unrelated to experienced detachment over time. Crafting for detachment may act like a coping strategy which activates under heavy job demands or stress but may not always be adaptive in increasing detachment [see also Patry et al. (2007) on avoidant leisure coping and Shimazu and Schaufeli (2007) on distraction coping].

OJC was positively related to life satisfaction (Studies 2–4), family role performance (Studies 3–4), and perceived work ability (Studies 3–4) over time. Moreover, small positive relationships from OJC to job satisfaction (in Study 2, but not in Studies 3–4) and work engagement (in Studies 2–3, but not in Study 4) were found. Thus, Hypothesis 3 was partially supported. It seems that OJC can help optimize well-being and performance in the off-job domain by helping workers to feel more satisfied with their life and to be more efficient in accomplishing their daily tasks, and in the work domain by enriching the personal resources, such as autonomy and mastery, that sustain work ability. OJC had more significant relationships to optimal functioning in German-speaking countries (Study 2) and Finland (Study 3) than in Japan (Study 4). The little time available to Japanese workers for crafting their off-job lives (average weekly working hours were 48.3 in this sample) may have diminished the positive effects of OJC for their optimal functioning. However, potential power issues due to the smaller sample size in Study 4 may also have contributed to the higher number of null relationships found among Japanese workers. To summarize, in general OJC has positive lagged effects especially to optimal functioning in the off-job domain, as well as to work ability over time in the job domain, suggesting spillover processes between OJC and outcomes in the work domain.

The results on criterion validity evidence (Studies 2–4) showed that crafting for meaning and crafting for affiliation had the most consistent positive relationships to optimal functioning across studies (see also Kujanpää et al., 2021). These dimensions were also the only ones that were positively related to optimal functioning among Japanese workers (Study 4). The literature on meaning-making and relational crafting supports the idea that focusing on creating more opportunities for experiencing meaning and affiliation could be a direct way of creating more optimal functioning (e.g., Rofcanin et al., 2019; Russo-Netzer, 2019; Chen et al., 2022).

### Convergent and incremental validity evidence

Hypothesis 4 received partial support, as the six OJC dimensions were mostly positively related to job crafting, proactive personality, and home crafting. All OJC dimensions were positively related to leisure crafting. To summarize, the results suggest that the experiences associated with “passive recovery” or “avoidance crafting” (Ten Brummelhuis and Trougakos, 2014; de Bloom et al., 2020), such as crafting for detachment and relaxation, may be crafted to gain a sense of rest and recovery especially in stressful life circumstances, whereas workers with more proactive personality engage more in “active” and challenging types of crafting such as crafting for autonomy, mastery, meaning, and affiliation.

Hypothesis 5 received partial support, since OJC was a significant contributor to all optimal functioning outcomes beyond proactive personality and leisure crafting (Study 3) as well as to life satisfaction, family role performance, and perceived work ability beyond home crafting and job crafting (Study 5). These results are in accordance with our conceptualization of OJC as a multidimensional phenomenon that includes crafting efforts and benefits in various off-job life domains beyond leisure (e.g., voluntary work and house- and childcare). For home crafting, it seems that OJC has a comparable utility in predicting optimal functioning at work, whereas OJC predicts additional variance in non-work outcomes compared to home crafting. Moreover, the strong internal consistency of the NOCS is a strength compared to the home crafting scale. Similarly, OJC predicts variance in life satisfaction and family role performance, but also in perceived work ability beyond job crafting. Overall, these results demonstrate that measuring crafting in different life domains based on a coherent needs-based theoretical framework is warranted to capture a fuller range of positive effects of crafting efforts.

### Discriminant validity evidence

OJC could reliably be distinguished from recreational activities (Study3), supporting Hypothesis 6. Aligning with theory, crafting one’s off-job life does not entail merely taking part in recreational activities, but requires proactivity and consideration of personal goals and need discrepancies (de Bloom et al., 2020).

### Theoretical contributions

Our study integrated the existing literature on leisure crafting, home crafting, job crafting, and psychological needs satisfaction to advance the so far scarce literature on crafting in the off-job domain. The concept of needs-based OJC integrates previously studied phenomena (job crafting, leisure crafting, home crafting), as well as life domains for which crafting has not been studied before, such as voluntary work and work breaks. The NOCS provides a highly reliable, valid and flexible instrument for measuring crafting in the off-job domain that has incremental value beyond proactive personality and leisure, home, and job crafting. In addition to a new instrument, this study was the first to explore differences in crafting in the off-job domain across various countries, showing that the posited needs-based OJC framework is valid in various cultures and can explain the processes through which crafting enhances optimal functioning (e.g., OJC for affiliation may lead to better family role relational performance through an increased sense of relatedness and family engagement), and why crafting one’s off-job life might be beneficial for optimal functioning in the job domain (e.g., increased needs satisfaction through OJC could translate into more work engagement through increased energy and positive mood).

### Limitations and future research

This study was not without limitations. First, our samples differed from each other in the distribution of the workers’ ages, genders, and professions. Thus, the differences observed in OJC between countries could also be due to the effects of demographic variables on OJC and its outcomes. In a similar vein, we did not conduct *a priori* power analyses, because there was limited existing evidence available concerning the anticipated effect sizes (for an insightful discussion on this challenge, see Weigelt et al., 2022). Our resources (i.e., financial, time, connections to companies) determined the maximum sample sizes we were able to realize. However, we aimed at and achieved similar or even bigger sample sizes as earlier validation studies focusing on job crafting, making us feel confident about the findings of our studies.

Moreover, we did not examine possible moderator effects in this study, which could make OJC more or less beneficial for the workers depending on their age, gender or profession (e.g., see Kooij et al., 2017 on age and job crafting). Second, we focused on crafting efforts and needs satisfaction as well as optimal functioning, but we did not take into account the role of motivational antecedents (“needs-as-motives,” Sheldon, 2011) of OJC. For example, OJC may be more beneficial over time for people who are more motivated (e.g., have a higher needs discrepancy) to engage in crafting (de Bloom et al., 2020). Qualitative studies, ecological momentary assessments and experimental studies could be applied to arrive at a better understanding of crafting episodes over time and factors which trigger or hinder crafting efforts. Third, future studies could examine the relationship between OJC and other types of crafting (e.g., job crafting) in more detail. Developing and validating a needs-based job crafting scale aligning with the identity-based integrative needs model of crafting (de Bloom et al., 2020) would be a logical next step in further elucidating how crafting processes play out across different life domains. Fourth, we measured only positive outcomes (well-being, family performance, and needs satisfaction). Since increased DRAMMA needs satisfaction may have even stronger effects on ill-being than on well-being (Kujanpää et al., 2020), future studies could examine whether it is possible to reduce ill-being through OJC. Finally, based on the Integrative Needs Model of Crafting (de Bloom et al., 2020), which posits crafting as mid- or long-term changes rather than incidental events, we used a relatively long time period between the measurements in Studies 2 to 4 (3 months). This means that possible shorter-term effects, such as day- or week-level effects may have been missed. Studies using diary-level methods could give insights into the fluctuations and possible short-term effects of OJC.

Due to space restrictions, we did not further consider the relationships between background variables (e.g., age, gender, education or working hours) and OJC. These relationships are worth looking into in future studies. Furthermore, despite the large number of studies on job crafting presented since the early 2000s, cross-cultural studies on crafting are rare (for exceptions, see Gordon et al., 2015; Yepes-Baldo et al., 2018). More careful examinations on the combined contextual effects of cultural, demographical, personal, and organizational characteristics on crafting could yield important insights into the applicability of the crafting concepts outside the “WEIRD” (western, educated, industrialized, rich and democratic; Henrich et al., 2010) countries.

Besides these broader outlines for future research, our results suggest that some OJC dimensions might produce needs satisfaction faster than others. It would be interesting to examine the combined effects or interactions of different crafting dimensions, and to explore possible hierarchical patterns in crafting (e.g., whether some crafting dimensions usually precede other dimensions chronologically). Besides linear relationships, curvilinear relationships between OJC and optimal functioning could be investigated in future studies (see also Shimazu et al., 2016).

### Practical implications

The concept of needs-based OJC, and the results of this research are salient to the health and well-being of workers facing increased intensification and time pressures in their work and decreased work-life balance. Through OJC dimensions such as OJC for meaning and for affiliation, workers can take a bottom-up, proactive approach to making changes in their off-job lives to enhance their needs satisfaction and optimal functioning, such as life satisfaction, perceived work ability, and performance in family roles. Even though focusing on recreational activities conducive to worker well-being is important, workers may prefer certain activities over others or have limited opportunities for taking part in a specific activity. In individual and group interventions aiding workers in shaping their off-job lives the focus should be more on the workers’ personal needs and goals, rather than focusing on the specific activities each worker might be engaging in as a part of their OJC efforts. For example, occupational health practitioners could encourage their clients to develop action plans to craft for need discrepancies that a client experiences to be the most salient for their well-being (Kujanpää, 2022). Similarly, arts and sports educators could use the six dimensions of needs-based OJC to encourage their students to reflect on potential ways to shape their hobbies to feel more personally satisfying.

It is also important to note that despite the considerable focus given to individual efforts in research on proactive behaviors, encouraging workers to craft their off-job time is no substitute for the responsibilities of organizations to ensure that their workers have sufficient off-job time and enough work-related resources to maintain a healthy work-life balance (Bal and Dóci, 2018). Managers could encourage their workers to achieve a healthy work-nonwork balance and promote an organizational culture wherein proactive shaping of personal and work schedules to match with one’s own needs is viewed as a positive resource that supports sustainable working lives within the organization.

In addition, the lagged effects found suggest that crafting can have effects which take time to fully manifest (e.g., several months). Thus, persistent crafting efforts over longer periods seem to yield the best rewards for well-being in the long run. The results suggest that especially crafting for meaning and for affiliation can be beneficial for optimal functioning. Furthermore, the concept of needs-based OJC is relevant not only to workers, but also to unemployed or retired individuals, students or hobbyists who might benefit from crafting their off-job lives to increase their needs satisfaction.

## Conclusion

In this study, we further developed the concept of needs-based OJC and validated a scale to measure the construct to lay bare its relationships to optimal functioning. We found evidence for structural validity over time in different countries and working contexts (three German-speaking countries, Finland and Japan). Across various cultures, people could provide concrete examples of how they craft their off-job time and reported that crafting benefits their well-being and performing daily tasks. In line with theory, OJC was positively related to job crafting, proactive personality, leisure crafting, home crafting, and needs satisfaction and distinct from recreational activities. OJC also explained additional variance beyond existing constructs and had positive relationships to life satisfaction, family role performance and work ability over time in three studies, and also small positive relationships to job satisfaction and work engagement over time. Needs-based OJC can benefit optimal functioning over time, and can be helpful for workers in addressing the demands imposed by an intensifying working and private life. Summing up, needs-based crafting can complement and enrich the existing body of literature on demands- and resources-based crafting by providing an overarching framework of needs which can be proactively addressed in various life domains.

## Data availability statement

The raw data supporting the conclusions of this article will be made available by the authors, without undue reservation.

## Ethics statement

Ethical review and approval were not required for the study on human participants in accordance with the local legislation and institutional requirements. The patients/participants provided their written informed consent to participate in this study.

## Author contributions

MK, LT, UK, AM, AS, and JdB contributed to the conception and design of the study. MK, AS, CW, RB, GB, PK, HT, and JdB collected the data. MK and CS performed the statistical analyses. MK, CS, and JdB wrote the first draft of the manuscript. All authors contributed to the article and approved the submitted version.

## Funding

This work was supported by the Academy of Finland under Grant [number 434 485] awarded to JdB. The University of Zurich Foundation supported data collections in Austria, Germany, and Switzerland and the contribution of GB and PK.

## Conflict of interest

The authors declare that the research was conducted in the absence of any commercial or financial relationships that could be construed as a potential conflict of interest.

## Publisher’s note

All claims expressed in this article are solely those of the authors and do not necessarily represent those of their affiliated organizations, or those of the publisher, the editors and the reviewers. Any product that may be evaluated in this article, or claim that may be made by its manufacturer, is not guaranteed or endorsed by the publisher.

## Appendix 1

Crafting constructs, recreational activities, their characteristics and distinguishing features

**Table:**

Construct	Definition and main focus	Theoretical basis	Motivation included in measurement?	Proactivity (= purposeful behaviors)	Life domains included	Empirical evidence of criterion validity
Needs-based (off- job) crafting	Goal-directed initiation of and engagement in crafting efforts intended to satisfy psychological needs	Identity-based integrative needs model of crafting (de Bloom et al., 2020)	Yes: Psychological needs (detachment from work, relaxation, autonomy, mastery, meaning, affiliation)	Yes, key to definition	Off-job life (i.e., leisure, voluntary work, house- and childcare, work breaks)	Yes. See this manuscript.
Job crafting according to Wrzesniewski and Dutton (2001)	Changing… 1) cognitive-, 2) task- and 3) relational boundaries of work to alter work meanings and identity	Integration of various theories (i.e., job design theories, social information processing, role innovation theory, role making)	Yes: Mix of goals and needs (i.e., goal setting, human connection, learning, personal development)	Yes, key to definition	Work	Yes. See review by Zhang and Parker, (2019)
Leisure crafting	Reshaping cognitive, task- and/or relational boundaries of leisure	Berg et al.’s (2010) work on unanswered callings (i.e., leisure crafting to fulfill unanswered callings) Wrzesniewski and Dutton (2001) model of job crafting	Yes: Mix of goals and needs (i.e., goal setting, human connection, learning, personal development)	Yes, key to definition	Leisure	Yes. Associated with meaning making, satisfaction of relatedness and autonomy needs (Petrou and Bakker, 2016; Petrou et al., 2017)
Job crafting according to Tims and Bakker (2010)	Lowering job demands and/or increasing job resources to achieve better person-job fit	Job design models (e.g., Hackman and Oldham, 1975 Demerouti et al., 2001)	No. Only behaviors are measured.	Yes, key to definition	Work	Yes. See review by Zhang and Parker, 2019
Home crafting	Seeking challenges and/or reducing demands at home for a meaningful life	Tims and Bakker (2010) model of job crafting	No. Only behaviors are measured.	Yes, key to definition	Home (i.e., house- and childcare)	No/not yet. Only predictors of home crafting have been examined
Recreational activities	Activities carried out during leisure time	None/scattered.	No.	No	Leisure	Not applicable. Associated with well-being and health (e.g., Pressman et al., 2009).

## Appendix 2

List of hypotheses tested in studies 1–5.

**Table:**

Hypothesis	Type of validity evidence examined	Hypothesis supported?
Explorative analyses	Structural validity evidence (a) 6-factor structure (b) Invariance over time (c) Internal consistency (d) Test–retest reliability	(a) Yes, 6-factor structure had a better fit than alternative models (Study 2) (b) Yes, invariant over time (Studies 2–4) (c) Yes, Cronbach’s α ranged between 0.70 and 0.90 across studies (Studies 2–5) (d) Yes, test–retest reliability ranged between 0.46 and 0.63 across time (Study 2)
*H1*: The number of examples for OJC efforts correlates positively with the matching scale dimension of the NOCS.	Criterion	Supported in Study 1b (except for OJC for detachment)
*H2*: OJC targeted at satisfying a DRAMMA need at T1 is positively related to the satisfaction of that need at T2 and T3.	Criterion	Supported in Study 3 (except for OJC for detachment)
*H3*: OJC at T1 is positively related to optimal functioning at T2 and T3; more specifically to (a) life satisfaction, (b) family role performance, (c) job satisfaction, (d) perceived work ability, and (e) work engagement.	Criterion	(a) Supported in Study 2; partially supported in Study 3 & 4 (b) Supported for task performance in Study 3; partially supported for task performance in Study 4 & relational performance in Study 3 & 4 (c) Partially supported in Study 2; not supported in Study 3 & 4 (d) Partially supported in Study 3 & 4 (e) Partially supported in Study 2 &3; not supported in Study 4
*H4*: OJC is positively related to (a) job crafting, (b) proactive personality, (c) leisure crafting, and (d) home crafting.	Convergent	(a) Partially supported in Study 5 (b) Partially supported in Study 3 (c) Supported in Study 3 (d) Partially supported in Study 5
*H5*: OJC predicts variance in optimal functioning beyond (a) job crafting, (b) proactive personality, (c) leisure crafting, and (d) home crafting.	Incremental	(a) Partially supported in Study 5 (b) Supported in Study 3 (c) Supported in Study 3 (d) Partially supported in Study 5
*H6*: Engaging in recreational activities is distinct from OJC.	Discriminant	Supported in Study 3

Study 1b = US participants (*n* = 97), Study 2 = German, Austrian, and Swiss participants (*n* = 2,104), Study 3 = Finnish participants (*n* = 578), Study 4 = Japanese participants (*n* = 228), Study 5 = UK participants (*n* = 237).
